# Climatic Aridity Gradient Modulates the Diversity of the Rhizosphere and Endosphere Bacterial Microbiomes of *Opuntia ficus-indica*

**DOI:** 10.3389/fmicb.2020.01622

**Published:** 2020-07-28

**Authors:** Fatma Karray, Mahmoud Gargouri, Asma Chebaane, Najla Mhiri, Ahmed Mliki, Sami Sayadi

**Affiliations:** ^1^Laboratory of Environmental Bioprocesses, Centre of Biotechnology of Sfax, Sfax, Tunisia; ^2^Laboratory of Plant Molecular Physiology, Centre of Biotechnology of Borj Cedria, Hammam-Lif, Tunisia; ^3^Center for Sustainable Development, College of Arts and Sciences, Qatar University, Doha, Qatar

**Keywords:** *Opuntia ficus-indica*, bacterial and archaeal microbiome, aridity gradient, rhizosphere soil, endosphere root, co-occurrence network

## Abstract

Recent microbiome research has shown that soil fertility, plant-associated microbiome, and crop production can be affected by abiotic environmental parameters. The effect of aridity gradient on rhizosphere-soil (rhizosphere) and endosphere-root (endosphere) prokaryotic structure and diversity associated with cacti remain poorly investigated and understood. In the current study, next-generation sequencing approaches were used to characterize the diversity and composition of bacteria and archaea associated with the rhizosphere and endosphere of *Opuntia ficus-indica* spineless cacti in four bioclimatic zones (humid, semi-arid, upper-arid, and lower-arid) in Tunisia. Our findings showed that bacterial and archaeal cactus microbiomes changed in inside and outside roots and along the aridity gradient. Plant compartment and aridity gradient were the influencing factors on the differentiation of microbial communities in rhizosphere and endosphere samples. The co-occurrence correlations between increased and decreased OTUs in rhizosphere and endosphere samples and soil parameters were determined according to the aridity gradient. *Blastococcus*, *Geodermatophilus*, *Pseudonocardia*, *Promicromonospora*, and *Sphingomonas* were identified as prevailing hubs and were considered as specific biomarkers taxa, which could play a crucial role on the aridity stress. Overall, our findings highlighted the prominence of the climatic aridity gradient on the equilibrium and diversity of microbial community composition in the rhizosphere and endosphere of cactus.

## Introduction

Semi-arid and arid zones occupy nearly 40% of the world’s terrestrial surface and are the most vulnerable to climate changes (Schlaepfer et al., 2017; Canter, 2018). These zones are characterized by high temperatures and low precipitations, and they are probably the most threatened by desertification. Abiotic stress, including high and low temperature, water deficit (drought) and flooding, salinity, UV radiation, light, and poor nutrient soils, have a significant impact on soil fertility and subsequently on agricultural production in semi-arid and arid regions (Shahid and Al-Shankiti, 2013; Cuevas et al., 2019; Hussain et al., 2019).

Cacti (*Cactaceae*) represent one of the most xerophyte plants that have their origin in arid and semi-arid ecosystems of central Mexico and the American continent (Hernandez-Hernandez et al., 2014). *Cactaceae* are an extremely diverse family of plants among which the genus *Opuntia* is the most numerous (Hernandez-Hernandez et al., 2014). Several morphological and physiological adaptations have been developed by the genus *Opuntia* in arid environments where water is the main factor limiting the development of most plant species. Prominent among these adaptations is the Crassulacean acid metabolism (CAM), a water-efficient type of photosynthesis, which allows plants to fix carbon dioxide through the night and prevent water loss during the day (Nobel, 2010). Besides, their morphologies are characterized by succulent bodies, thick epidermis of cladodes covered with spines, and shallow and extensive root systems that enable cacti to exploit scarce rainfall and high temperatures. These physiological and biochemical strategies developed by cacti have permitted them to resist and spread under conditions of high temperatures and scarce and erratic rainfall (Nefzaoui et al., 2010, 2014). They play a significant role in the protection of local fauna in arid ecosystems. Moreover, plantations of cactus for fruit and forage production have been developed in all continents (Nefzaoui et al., 2010). The increasing interest in cactus cultivation, particularly *Opuntia ficus-indica*, is due to its implication in the future achievement of sustainable agriculture in arid and semi-arid zones. It is widely documented that a significant diversity of microbes associated with plants form a biological unit named “holobiont” (Bordenstein and Theis, 2015; Vandenkoornhuyse et al., 2015). These microorganisms can affect host fitness, development, adaptation, survival, and distribution of plants. Several previous studies, based on culture-dependent and culture-independent approaches, reported that bacteria and fungi live in different plant compartments, and some of them are involved in promoting plant growth and/or tolerance to abiotic stress in arid ecosystems. Numerous factors influencing the plant microbiome, such as the host genotype, the physical and chemical properties of soil, the seasonal variations, the agricultural practices, the plant compartment, the biogeography of the plant species, and the plant development, have been studied using high-throughput sequencing technologies (Coleman-Derr et al., 2016; Fonseca-García et al., 2016; Eida et al., 2018; Lee et al., 2019; Zheng and Gong, 2019). However, despite many studies carried out using culture and uncultured-based methods to characterize cactus-associated microbiome in arid zones, the effect of aridity on microbiome assembly has not been fully explored. Several studies have cataloged the endophytic bacteria and fungi colonizing intercellular spaces in various internal plant tissues of cactus species in different countries (Bezerra et al., 2017). Besides fungal endophytes, endophytic bacteria and bacteria on the rhizoplane of cacti from the desert areas in Mexico have been described by other studies, revealing the effect of the association of bacteria, archaea, and fungi on seed germination and cactus persistence in their natural environment (Puente et al., 2004a, b, 2009a,b; Fonseca-García et al., 2016; Bezerra et al., 2017). Cactus endophytic plant growth-promoting bacteria (PGPR) are known to facilitate plant growth by promoting production of phytohormones (auxins, gibberelins, cytokinins, and ethylene), diazotrophic fixation of nitrogen, and solubilization of phosphate. Moreover, they enhance the plant’s tolerance to various stresses, such as high salinity, drought, metal toxicity, pesticide load, and activity against a broad spectrum of phytopathogens (Puente et al., 2009a; Lopez et al., 2011; Kavamura et al., 2013). Two rhizobacteria related to the genus *Bacillus* were isolated from Brazilian cacti exhibiting plant growth-promotion abilities under drought conditions (Kavamura et al., 2013).

In North Africa, particularly in Tunisia, wild and cultivated populations of *Opuntia ficus-indica* (Cactaceae) exist from north to south in all bioclimatic zones, from the humid to the semi-arid zones (Nefzaoui and Salem, 2001). It is mainly cultivated for fruit production and forage, particularly in upper-arid regions (Nefzaoui et al., 2010). The investigation on microbial communities and the mechanisms by which particular bacteria associated with cactus plants induce abiotic stress tolerance in plants is still poorly understood. The main focus of the present research was to characterize the prokaryotic (bacteria and archaea) community’s diversity and composition in *Opuntia ficus-indica* using DGGE and high-throughput sequencing (Illumina MiSeq) approaches. The impact of the aridity gradient and soil physical and chemical properties on the diversity and the composition of the prokaryotic communities associated with spineless cacti were evaluated.

## Materials and Methods

### Sampling

Prickly pear trees (*Opuntia ficusindica f. inermis*) growing in wild areas protected by the forest administration in Tunisia were selected for this study: rhizosphere soil (5 mm of soil adhering to the root surface) and root endosphere (the interior of the root). The studied locations were chosen to represent an increased aridity gradient characterized by a decreased total annual precipitation and mean temperature ranging from 1200 to 100 mm (Verner et al., 2018) and from 18 to 20.2°C (meteorological stations in Tunisia), respectively, and covering four bioclimatic zones (humid, semi-arid, upper-arid, lower-arid) (Table 1). The latitude and longitude for each site were noted using a GPS device during the field work (Table 1). Roots collected from cactus plants aged between 3 and 4 years were considered as young wild plantations. The height of cactus plants is about 3–3.5 m. After digging until the first primary root appeared, the lateral fine roots were collected. Both rhizosphere soil samples and approx. 30 g of roots were collected early March 2015 from healthy plants. All samples were collected in sterile bottles, transported in cold conditions to the laboratory, and kept aseptically at −80°C until analysis. At each sampling site, three to five distinct replicates were collected. To avoid any contamination with left root fragments, rhizospheric samples were sifted by a 2 mm sieve to remove all rocks, roots, and large debris. Collected root samples were washed by shaking during 30 min in sterilized water after a 2 min wash in 1% sodium azide to eliminate epiphytic microoganisms.

**TABLE 1:** Geographic positions and annual precipitation of selected *Opuntia ficus-indica* plantation fields.

						**GPS coordinates**	
**Bioclimatic stage**	**Annual precipitation (mm)**	**Annual mean temperature (°C)**	**Altitude (m)**	**Sample name**	**Sample location name**	**Latitude**	**Longtitude**
Humid	800–1200	18	<10	BZ	Bizerte	37.1227	9.54539
				BA1		36.5056	10.5222
Semi-arid	400–600	19.5	90–150	BA2	Bou Argoub	36.5073	10.4164
				BA3		36.5056	10.4322
				KA1		35.3806	9.3004
		20	400–600	KA2	Kairouan	35.3942	9.2859
				KA3		35.4008	10.005
				KS1		35.2112	8.4806
				KS2		35.2111	8.4750
Upper-arid	200–400	19	900–1000	KS3	Kasserin	35.2223	8.4631
				KS4		35.2234	8.4632
				KS5		35.2244	8.4632
				KS6		35.2921	8.6621
				SB1		35.3123	9.2129
		20	200–400	SB2	Sidi Bouzid	35.1719	9.2701
				SB3		35.3123	9.2701
				GF1		34.1504	9.1716
Lower arid	100–200	20.2	100–150	GF2	Gafsa	34.1502	9.1713
				GF3		35.5547	10.1754

**Rhizosphere and endosphere samples were collected along an increased aridity and passed through four bioclimatic stages ranging from humid to lower-arid sites.**

### Soil Measurements

Soil properties were measured as previously described (Bao, 2000), including total organic carbon (TOC) following the method of Walkley (1947), total nitrogen (TN) using the modified Kjeldahl procedure (Liao, 1981), and total soil phosphorus (TP) using the Mehlich method (Föhse et al., 1991), and the ionic contents of potassium (K^+^), calcium (Ca^2+^), and iron (Fe^3+^) were determined using the atomic absorption method (Lindsay and Norvell, 1978). Soil pH was measured at a water-to-soil mass ratio of 1:1 using a pH meter with an adjusted combined glass electrode (FE20, Mettler-Toledo Instruments, China). Soil moisture (MO) was determined gravimetrically by weighing after drying in an oven at 105°C for 10 h (Craze, 1990).

### DNA Extraction

MO BIO PowerSoil DNA Isolation Kit (MO BIO Laboratories) was used to extract total community DNA from 250 mg pellet of rhizosphere and powdered endosphere roots according to the manufacturer’s protocol. Subsequently, all DNAs were quantified using NanoDrop System (2000; Thermo Fisher Scientific).

### PCR-DGGE and Sequence Analysis

The V3–V5 hypervariable regions of the 16S rRNA gene were amplified using a bacteria-specific primer set (341FGC/907rR, Muyzer et al., 1993, 1995). The PCR products were analyzed by denaturing gradient gel electrophoresis (DGGE) using the DCode multiple system (Bio-Rad). DGGE was carried out as previously described using a denaturing gradient of 35–65% denaturants in 6% (w/v) polyacrylamide gel (BenAbdallah et al., 2016). Predominant DGGE bands were excised, reamplifed, and sequenced. Sequences and phylogenetic analysis of the rRNA 16S sequences were performed as previously reported (BenAbdallah et al., 2018).

### Illumina Sequencing and Data Processing

Sequencing of PCR amplicons of 16S rDNA was conducted with the Illumina MiSeq platform (CBS, Sfax, Tunisia) targeting the V3–V4 hypervariable regions using prokaryotic universal (Pro341/Pro805R) primer sets (Takahashi et al., 2014) for 2 × 300 bp paired-end sequencing (Illumina). The amplification of PCR products and Illumina library preparations were achieved as previously reported (BenAbdallah et al., 2018). Raw data acquired from the Illumina MiSeq sequencing platform were analyzed using the QIIME software package 1.9.1 (Caporaso et al., 2010) as previously described (BenAbdallah et al., 2018). Plant chloroplast and mitochondrial OTUs were removed from all sequences samples.

### Statistical Analysis

Significant differences between physicochemical parameters of soil across the different bioclimatic zones were calculated using the HSD–Tukey test. The relative abundance of each phylum, class, and order in different rhizosphere and endosphere samples was summarized in histogram graphs using R software environment (v.3.2.5) (R Core Team, 2017), and the significant differences were determined using HSD Tukey statistical test. A Venn diagram was constructed using the package Venn Diagram in R (v 3.2.5). α*-diversity indices* (Simpson and number of observed OTUs) were determined after normalizing to a depth of 1000 reads and were calculated by QIIME (Caporaso et al., 2010) using ANOVA test analysis. Differences in β-diversity between the samples were tested through permutational multivariate analysis of variance (PERMANOVA, 5000 permutations) and visualized using non-metric multidimensional scaling (Langfelder and Horvath, 2012). All heat maps were drawn by the “aheatmap” function in the “NMF” package of R^1^. Network analysis was performed on sample OTUs and soil properties. Prior to analysis, OTUs were classified based on their abundances whether increased or decreased, following strictly (> or < 0.9) the aridity gradient in the endosphere and rhizosphere samples, using the humid relative abundance data as a reference. Co-occurrence of OTUs was defined based on their Pearson/Spearman correlations using the WGCNA package (Oksanen et al., 2013). The node shapes represent increased or decreased OTUs in the endosphere or rhizosphere; the node was colored by taxonomy, and the edges connecting the nodes represent correlations between OTU pairs and soil proprieties. Negative and positive co-occurrence relationships based on strength of correlation at *r* ≤ −0.9 and ≥ 0.9 and *p* ≤ 0.05. All *P-*values were adjusted for multiple testing using the Benjamini and Hochberg FDR controlling procedure (Benjamini et al., 2006). Networks were created and visualized with the open source platform Cytoscape 3.7.1.

### Data Access

Bacterial sequence data of the DGGE bands has been submitted to GenBank, and the assigned accession numbers were from MK208464 to MK208475. 16S raw reads have been deposited in the Short Read Archive of NCBI under project no. PRJNA511384.

## Results

### Soil Physicochemical Analysis

The pH, soil water content, humidity%, organic matter%, nitrogen%, calcium, potassium, iron, phosphate, and capacity exchange cation (CEC) parameters were measured using standard soil analytic methods. Soil characteristics associated with spineless *Opuntia ficus-indica* are presented in Table 2 and Supplementary Figure S1. A significant difference in the parameter values or percentages between bioclimatic zones (humid, semi-arid, upper-arid, and lower-arid) was observed. For instance, Fe^3+^, K^+^, TP, and CEC concentrations and the% of humidity and TOC decreased while the Ca^2+^ concentration increased concomitantly with the aridity gradient (HSD Tukey test *p* < 0.05; Supplementary Table S1). pH and the total nitrogen were comparable in all bioclimatic zones (HSD Tukey test *p* < 0.05; Supplementary Table S1).

**TABLE 2:** Principal characteristics of soil samples of spineless *Opuntia ficus-indica* collected across the four bioclimatic zones (humid, semi-arid, upper-aid, and lower-arid).

**Bioclimatic stage**	**Sample name**	**pH**	**Humidity (%)**	**TP (mg/l)**	**TOC (%)**	**TN (%)**	**Fe^3+^ (mg/kg)**	**Ca^2+^ (mg/kg)**	**K^+^ (mg/kg)**	**CEC (mol/kg)**
Humid	BZ	74.3	3.91	13.01	4.44	0.45	20.86	4775.63	78.88	19.80
	BA1	7.88	4.06	22.70	1.72	0.39	18.58	1080.46	86.19	8.80
Semi-arid	BA2	6.85	0.08	5.14	0.26	0.70	17.85	1400.71	103.63	0.95
	BA3	7.36	2.07	13.92	0.99	0.545	18.21	1240.58	94.91	4.87
	KA1	6.68	4.31	1.30	0.52	0.46	11.58	2227.83	88.29	2.35
	KA2	6.65	6.05	13.33	1.34	0.56	12.21	2293.08	98.48	7.70
	KA3	6.61	2.56	3.48	0.47	0.52	11.58	2227.83	88.29	1.75
	KS1	5.74	2.08	1.49	0.60	1.41	13.26	2599.17	91.52	5.10
	KS2	7.82	6.52	1.14	1.69	1.56	12.93	3142.08	79.09	13.00
Upper-arid	KS3	7.61	1.52	1.21	0.54	0.70	16.01	1298.42	50.75	4.30
	KS4	6.42	1.81	1.00	0.74	0.88	13.95	1452.50	51.84	4.45
	KS5	6.46	2.13	1.10	1.39	0.72	12.93	3142.08	79.09	5.90
	KS6	6.74	1.47	0.72	0.59	0.40	16.01	1298.42	50.75	5.00
	SB1	8.19	6.81	2.15	1.21	0.51	14.53	4812.92	170.45	6.75
	SB2	7.99	3.82	1.88	0.95	0.65	14.34	6004.17	163.68	7.45
	SB3	8.00	8.87	0.92	1.44	0.46	14.53	4812.92	170.45	20.55
	GF1	8.22	4.24	3.16	2.24	0.61	17.36	4923.75	131.03	8.00
Lower arid	GF2	7.77	6.04	18.39	4.70	0.46	17.45	5740.42	274.63	11.50
	GF3	8.34	3.63	6.64	1.96	0.57	17.36	4923.75	131.03	7.75

**K*^+^, *Potassium ion; Ca^2+^, calcium ion; Fe^3+^, iron ion; TOC, pH, total organic carbon; TN, total nitrogen; TP, total phosphorus; H, humidity; CEC, cation exchange capacity.**

### Prokaryotic Diversity Profiles in the Endosphere and Rhizosphere of *Opuntia ficus-indica*

**FIGURE 1:**
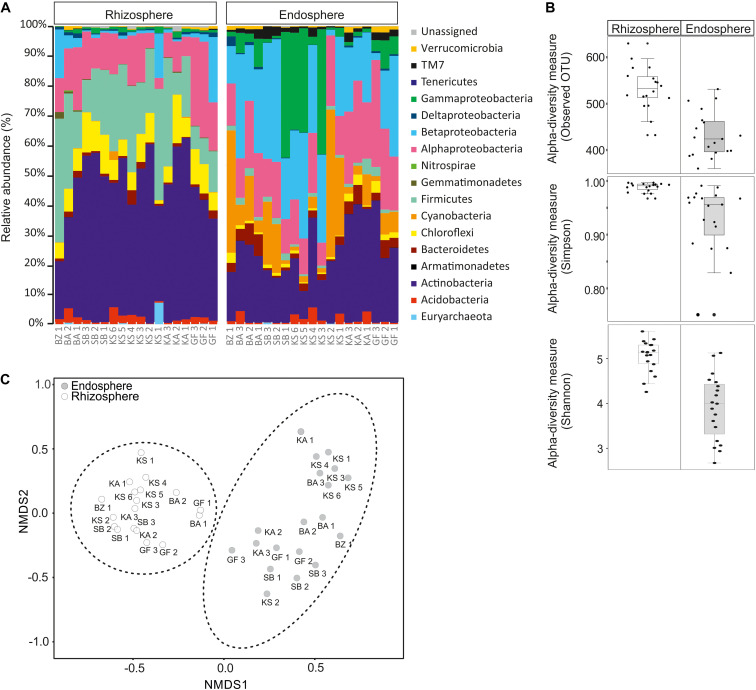
Relative abundance of rhizosphere and endosphere prokaryotic communities of *Opuntia ficus-indica.* **(A)** Relative phylogenetic abundance based on frequencies of 16S rRNA gene sequences affiliated with archaea and major bacterial phyla or proteobacterial classes. **(B)** Prokaryotic alpha diversity indices (Simpson: *p*-value: 0.0007; ANOVA test: *F*-value: 4.03; Shannon: *p*-value: 1.968 10^–06^; ANOVA test: *F*-value: 6.0511) and richness (observed OUT) (*p*-value: 3.44 10^–07^, ANOVA test: *F*-value: 6.26) in: rhizosphere soil (rhizosphere) and endosphere root (endosphere). **(C)** Non-metric multidimensional scaling (NMDS) plots for Bray–Curtis distances of the bacterial and archaeal communities associated with *Opuntia ficus-indica* in the rhizosphere and endosphere (stress value = 0.10, PERMANOVA, *P* < 0.001).

The composition and diversity of rhizosphere and endosphere prokaryotic communities were assessed by next-generation sequencing of 16S ribosomal RNA gene amplicons. After quality filtering and removal of potential chimera’s sequences, approximately 1,709,770 high-quality sequences with an average read length of 450 bp were obtained for further analyses. All sequences were classified at phylum level, except for proteobacterial groups, which were further divided by class (Figure 1A). Sequences obtained were assigned to thirteen bacterial phyla and one archaea. The global structure of the prokaryotic communities found from each sample is shown in Figure 1A. In rhizosphere samples, the bacteria domain was mainly represented by Proteobacteria (6.96–40.43%) followed by Actinobacteria (19.42–60.69%), Firmicutes (7.26–41.43%), Chloroflexi (0.3–16.22%), Acidobacteria (0.16–5.67%), Bacteroidetes (0.28–4.82%), Cyanobacteria (0.01–2.92%), Gemmatimonadetes (0.04–2.23%), and Verrucomicrobia (0.06–1.28%). In the Proteobacteria phylum, Alphaproteobacteria (5.09–32.15%) and Betaproteobacteria (0.43–21.86%) are clearly dominating in all samples, followed by Gammaproteobacteria (0.18–2.08%) and Deltaproteobacteria (0.16–1.56%) classes. Other phyla with low abundances (<1% of all sequences) were Nitrospirae (0–0.41%), TM7 (0.006–0.41%), Armatimonadetes (0–0.06%), and Tenericutes (0–0.04%) (Figure 1A). Proteobacteria (26.29–82.48%), Cyanobacteria (1.22–49.46%), Actinobacteria (9.40–40.97%), Bacteroidetes (0.55–7.28%), Acidobacteria (0.006–5.63%), Firmicutes (0.43–5.22%), TM7 (0.42–4.25), Chloroflexi (0.30–3.91%), and Verrucomicrobia (0.07–2.05%) represent the major phyla in endosphere samples. Proteobacteria, the dominant phylum, was divided into four classes: Gammaproteobacteria (0.69–42.10%) followed by Betaproteobacteria (2.28–41.10%), Alphaproteobacteria (7.70–35.89%), and Deltaproteobacteria (0.08–3.24%). Tenericutes (0–0.11%), Armatimonadetes (0–0.09%), and Gemmatimonadetes (0–0.05%) were detected in lower abundance (<1% of all sequences, Figure 1A). Within the domain *Archaea*, a low relative abundance in the rhizosphere (0–7.22%) and endosphere (0–0.56%) were retrieved. Only six OTUs, belonging to the classes Halobacteria and Methanomicrobia within the Euryarchaeota phylum were detected. Among Halobacteria, 1 and 2 OTUs were affiliated with the genera *Haloarcula* and *Halorubrum*, respectively.

Our results revealed that Proteobacteria, Actinobacteria, Firmicutes, Chloroflexi, Acidobacteria, Bacteroidetes, Cyanobacteria, and Verrucomicrobia dominated in rhizosphere and endosphere samples, whereas Gemmatimonadetes and TM7 were only abundant in the rhizosphere and endosphere, respectively. The comparison of the relative abundance between the rhizosphere and endosphere exhibited that Actinobacteria, Firmicutes, and Chloroflexi phyla were more abundant in rhizosphere samples than in endosphere samples. However, Cyanobacteria, Proteobacteria (especially Gammaproteobacteria, and Betaproteobacteria classes) and TM7 phyla were more dominant in endosphere than rhizosphere samples. Furthermore, rhizosphere samples displayed a higher prokaryotic richness (i.e., observed OUT, Figure 1B) and diversity (Simpson and Shannon indices, Figure 1B) than endosphere samples. All of these results suggest that the community composition was highly related to sample type (rhizosphere or endosphere). In order to identify community structure and co-occurrence patterns between different samples, a non-metric multidimensional scaling (NMDS) analysis based on the distribution of prokaryotic structure at OTU level was performed (Figure 1C). The NMDS analysis showed two distinct groups associated with rhizosphere and endosphere samples.

### The Prokaryotic Microbiome Diversity Structure of the Cactus Endosphere and Rhizosphere in the Different Bioclimatic Zones

In order to explore the effect of the aridity degree on the prokaryotic microbiome community structure and composition, DGGE and high-throughput sequencing of 16S rRNA genes analysis were investigated. DGGE profiles from rhizosphere and endosphere samples are presented in Supplementary Figure S2. Eight and four bands from rhizosphere and endosphere samples were excised, reamplified, and sequenced. The results of band DGGE sequences obtained from rhizosphere samples within the bacterial domain show that sequences were regrouped in the Firmicutes, Actinobacteria, and Proteobacteria (Betaproteobacteria class) phyla (Supplementary Table S2). All bands were closely related to the genera *Bacillus*, *Arthrobacter*, *Rubrobacter*, *Geodermatophilus*, and *Frankia* (95–99% similarity). In endosphere samples, all band DGGE sequences were clustered within Betaproteobacteria class and Actinobacteria phylum (Supplementary Table S2). *Burkholderia*, *Massilia*, *Streptomyces*, and *Amycolatopsis* genera were identified in endosphere samples.

**FIGURE 2:**
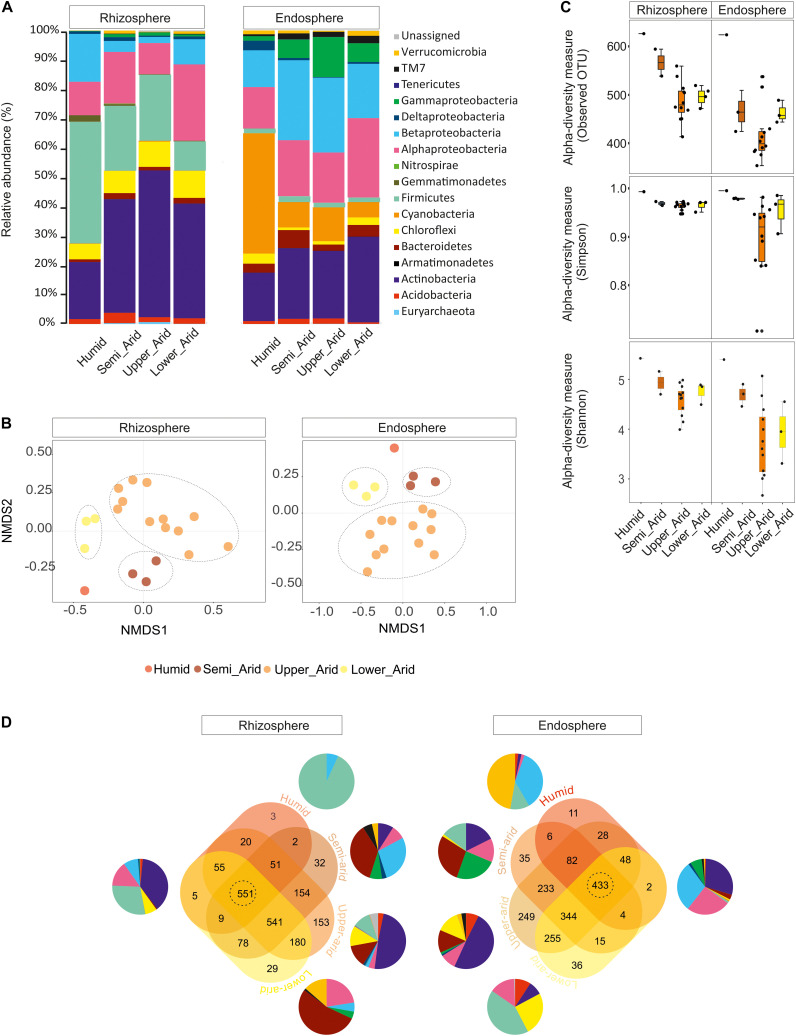
Relative abundance of rhizosphere and endosphere prokaryotic communities of *Opuntia ficus-indica* across the different bioclimatic zones (humid, semi-arid, upper-arid, lower-arid). **(A)** Relative phylogenetic abundance based on frequencies of 16S rRNA gene sequences affiliated with archaea and major bacterial phyla or proteobacterial classes. **(B)** Non-metric multidimensional scaling (NMDS) plots for Bray–Curtis distances of the prokaryotic communities associated with *Opuntia ficus-indica* across bioclimatic zones in the rhizosphere (stress value = 0.122, PERMANOVA, *P* < 0.001) and endosphere (stress value = 0.093, PERMANOVA, *P* < 0.03). **(C)** Prokaryotic alpha diversity indices (Simpson: *p*-value: 0.0024; ANOVA test: *F*-value: 4.24; Shannon: *p*-value: 1.9713 10^–05^, ANOVA test: *F*-value: 8.0451) and richness (observed OUT) (*p*-value: 3.13 10^–06^, ANOVA test: *F*-value: 9.83) in the rhizosphere and endosphere from each bioclimatic zone. **(D)** Venn diagrams showing the distribution of prokaryotic measurable OTUs associated with *Opuntia ficus-indica* in the rhizosphere and endosphere from each bioclimatic zone and shared OTUs as described for **(A)**. Samples from the humid, semi-arid, upper-arid, and lower-arid bioclimatic zones shared a high-degree overlap (551 and 433 OTUs) in the rhizosphere and endosphere, respectively. Specific number of OTUs for each bioclimatic zone and their taxonomic affiliation were changed across the aridity gradient. The higher numbers of OTUs (153 and 249 OTUs) were observed in the upper-arid bioclimatic zone in rhizosphere and endosphere samples, respectively.

**FIGURE 3:**
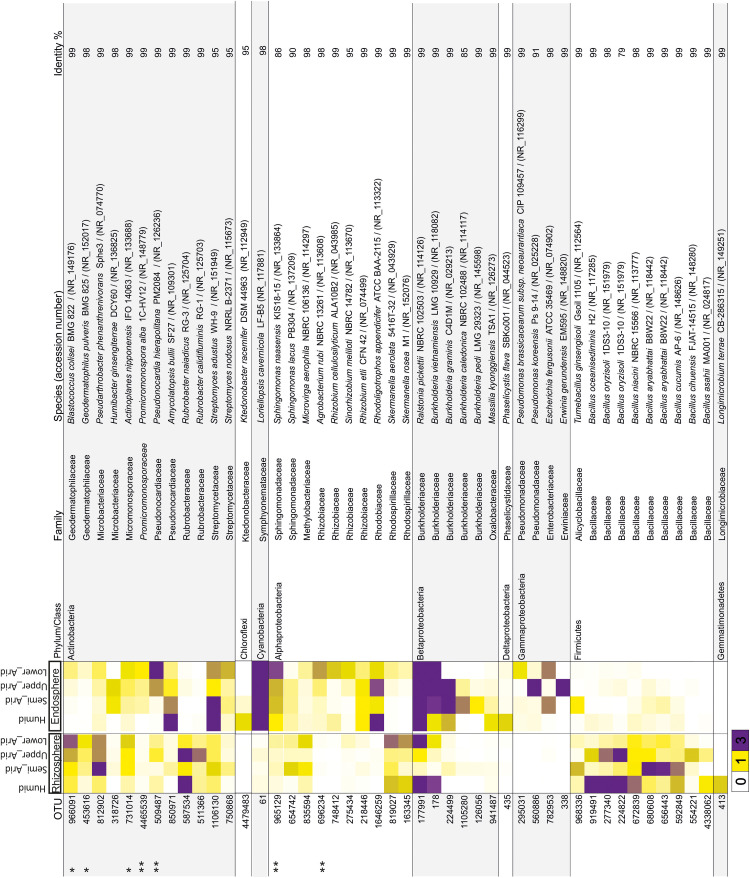
Heat map showing the relative abundance of dominant OTUs (>1% of all sequences) related to rhizosphere and endosphere samples from each bioclimatic zone. The OTU ID is noted to the left of the heat map while the annotation, given as the lowest taxonomic level based on NCBI database identity, is to the right of the heat map. The color intensity for each panel corresponds to the OTU abundance; white (0%) indicates low relative abundance, through yellow (>1%) to purple (> 3%) which indicate a high level of relative abundance. ^∗^Increased rhizosphere OTU, ^∗∗^increased endosphere OTU.

Using the high-throughput sequencing approach (MiSeq Illumina), the rRNA 16S sequences acquired from all samples were clustered according to their bioclimatic zones and analyzed at the phylum level (Figure 2A). The relative abundance of populations related to Alphaproteobacteria and Actinobacteria were significantly enriched along the aridity gradients in both rhizosphere and endosphere (HSD Tukey test *p* < 0.05; Supplementary Table S3). However, Betaproteobacteria and Verrucomicrobia were enriched in the endosphere and rhizosphere, respectively (HSD Tukey test *p* < 0.05; Supplementary Table S3). Firmicutes, Gemmatimonadetes, and Betaproteobacteria decreased along the aridity degree in the rhizosphere, and Cyanobacteria and Deltaproteobacteria decreased in the endosphere (HSD Tukey test *p* < 0.05; Supplementary Table S3). Our findings revealed that rhizosphere and endosphere prokaryotic microbiomes change across the climatic aridity gradient, which is supported by NMDS analysis based on the distribution of prokaryotic composition at the OTU level in each compartment (Figure 2B). Distinct groups associated with different bioclimatic zones were observed in the rhizosphere and endosphere. Alpha diversity (richness and diversity) of prokaryotic communities in all bioclimatic zones in both rhizosphere and endosphere samples was analyzed (Figure 2C). Our results showed that the semi-arid bioclimatic zone presents the highest richness in the rhizosphere and endosphere. In the rhizosphere, the diversity (Simpson and Shannon indices) of prokaryotic communities was similar among all bioclimatic zones. In the endosphere sample, the highest diversity was observed in the lower-arid zone, the most arid zone. The highest diversity was observed in the humid zone whatever the sample (rhizosphere or endosphere). Prokaryotic assembled analysis along the aridity gradient at the OTU level was investigated. Samples from humid, semi-arid, upper-arid, and lower-arid bioclimatic zones shared a high-degree overlap (551 and 433 OTUs) in rhizosphere and endosphere samples, respectively (Figure 2D). The Venn diagram showed specific number of OTUs for each aridity zone, and their taxonomic affiliation changed following the aridity gradient as presented in Figure 2D. The higher numbers of OTUs were observed in the upper-arid bioclimatic zone in rhizosphere and endosphere samples, respectively (153 and 249 OTUs). Their representative OTU sequences were mainly related to the Actinobacteria phylum. To get better insight into the differences of the dominant prokaryotic community across the five different bioclimatic zones in the rhizosphere and endosphere, we applied heat map analyses of the most abundant OTUs (>1% of all sequences), which highlighted their relative distributions and abundances (Figure 3). Interestingly, among the abundant OTUs (>1%), the rhizosphere was dominated by twenty-five abundant OTUs, affiliated to Alphaproteobacteria, Betaproteobacteria, Actinobacteria, Firmicutes, and Gemmatimonadetes phyla (Figure 3). Furthermore, twenty-eight representative OTUs related to Proteobacteria (Alpha, Beta, Gamma, and Delta), Actinobacteria, Cyanobacteria, and Chloroflexi phyla (Figure 3) were detected in endosphere samples. These dominant OTUs belonging to genera *Sphingomonas*, *Ralstonia*, *Burkholderia*, *Pseudarthrobacter*, *Actinoplanes*, *Pseudonocardia*, and *Streptomyces* were detected in both rhizosphere and endosphere. Specific species associated with the rhizosphere were related to *Skermanella*, *Microvirga*, *Bacillus*, *Tumebacillus*, *Longimicrobium*, *Rubrobacter*, *Geodermatophilus*, and *Blastococcus* genera while *Amycolatopsis*, *Promicromonospora*, *Humibacter*, *Ktedonobacter*, *Loriellopsis*, *Sinorhizobium*, *Rhizobium*, *Agrobacterium*, *Rhodoligotrophos*, *Massilia*, *Phaselicystis*, *Pseudomonas*, *Escherichia*, and *Erwinia*, genera, which were only present in endosphere samples. Additionally, among these dominant OTUs, some rhizosphere or endosphere OTUs increased, in inside and outside roots, across the aridity gradient and became more and more abundant in the lower-arid, the most arid zone (Figure 3). Within Actinobacteria, three rhizosphere OTUs (966091, 453616, and 731014) and two endosphere OTUs (4465539 and 509487) were related to *Blastococcus*, *Geodermatophilus*, *Actinoplanes*, *Promicromonospora*, and *Pseudonocardia* genera, respectively. Moreover, two other endosphere OTUs (965129 and 696234) belonged to *Sphingomonas* and *Agrobacterium* within the Alphaproteobacteria class.

### Bacterial Correlation Networks of the Cactus Rhizospheric Under Increased and Decreased Aridity Degree

Bacterial OTUs, which could involve the increased and decreased OTUs following the aridity degree in both rhizosphere and endosphere samples, were classified and determined based on their relative abundance (>0.1% of all sequences) (Supplementary Table S4). Heat map analysis was applied to display all increased and decreased bacterial OTUs in rhizosphere and endosphere samples following the aridity gradient (Supplementary Figure S3). Interestingly, our results demonstrated that rhizosphere and endosphere samples exhibited forty-eight and fifty-two increased OTUs, respectively, among which seven OTUs were shared by both rhizosphere and endosphere samples. The increased OTUs were closely related to Actinobacteria, Bacteroidetes, Chloroflexi, Firmicutes, Alphaproteobacteria, and Betaproteobacteria. Furthermore, increased OTUs affiliated with Cyanobacteria and TM7 groups were identified in endosphere samples while one OTU related to unidentified prokaryotic microbe was recognized in the rhizosphere (Supplementary Figure S3 and Supplementary Table S4). Eight and seventeen decreased OTUs were revealed in the rhizosphere and endosphere, respectively. These OTUs were affiliated with Acidobacteria, Actinobacteria, Chloroflexi, Firmicutes, and Alphaproteobacteria. Moreover, the TM7 group was detected in rhizosphere samples while Bacteroidetes, Verrucomicrobia, and Cyanobacteria were observed in endosphere samples (Supplementary Figure S3 and Supplementary Table S4). In inside and outside roots (rhizosphere and endosphere), specific increased and decreased OTUs were found displaying the effect of aridity on the composition of rhizosphere and endosphere communities associated with spineless cacti.

**FIGURE 4:**
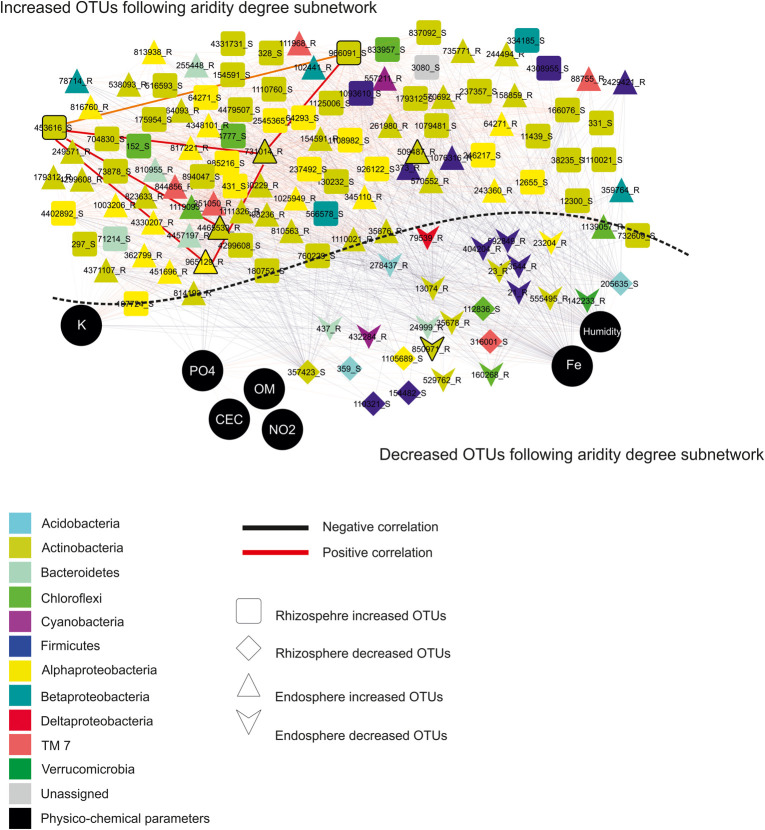
Network topology plots of *Opuntia* bacterial communities associated with the rhizosphere and endosphere: Increased OTUs following the aridity degree subnetwork and decreased OTUs following the aridity degree subnetwork. Nodes: physicochemical parameters are represented by black circles, square: rhizosphere**-**increased OTUs, diamond: rhizosphere**-**decreased OTUs, triangle: endosphere-increased OTUs, and heart: endosphere-decreased OTUs. Lines connecting two nodes represent significant correlations: red represents a positive correlation (co-presence), and black represents a negative correlation (mutual exclusion).

These findings allowed constructing co-occurrence patterns of potential interactions that could firstly occur between bacterial taxa related to increased and decreased OTUs following the aridity degree present in the rhizosphere and endosphere in spineless cactus and secondly with soil environmental parameters. Clearly, non-random co-occurrence patterns of bacterial communities were identified. Two subnetworks (the increased OTUs following aridity degree and the decreased OTUs following aridity degree subnetworks) constructed based on correlation were visualized on the network (Figure 4). The increased OTUs following the aridity degree subnetwork were characterized by a strong co-occurrence between increased OTU in rhizosphere and endosphere samples (Supplementary Figure S4 and Supplementary Table S5). These were later negatively correlated with potassium. The first subnetwork was negatively correlated with the second subnetwork (the decreased OTUs following the aridity degree subnetwork). The decreased OTU following the aridity degree subnetwork in rhizosphere and endosphere samples displayed a positive and robust interaction with iron, humidity, and phosphate. Positive correlation was also revealed between environmental parameters: nitrogen, phosphate, organic matter, and the cation exchange capacity of the soil. Iron and humidity were positively correlated whereas potassium was negatively correlated with them. The increased OTUs following the aridity degree subnetwork exhibited strong co-occurrence bacterial patterns mostly related to Actinobacteria and Alphaproteobacteria, followed by Firmicutes, Chloroflexi, Betaproteobacteria, Bacteroidetes, TM7, Cyanobacteria, and unidentified prokaryotes. The top twenty-two rhizosphere and endosphere OTUs with a high degree of connectivity (≥56) were designated (Table 3). Among them, five increased OTUs, previously identified as dominant OTUs and possessing a positive correlation and a strong interaction between them, were selected. Two rhizosphere-increased OTUs (966091 and 453616) were related to bacterial species *Blastococcus* and *Geodermatophilus*, and three endosphere-increased OTUs (731014, 4465539, and 965129) were affiliated with *Pseudonocardia*, *Promicromonospora*, and *Sphingomonas* genera (Table 3). Our results suggest that bacterial species related to these five hubs could play a major role on aridity stress.

**TABLE 3:** Top twenty-two bacterial hubs for the increased and decreased OTUs following the aridity gradient subnetworks associated with *Opuntia*.

	**OTU**	**Degree**	**Increased**	**Decreased**	**Taxonomy (phylum/class; family)**	**Species (accession number)**	**Similarity (%)**
Rhizopshere	328	89	x		Actinobacteria; Parviterribacteraceae	*Parviterribacter kavangonensis* D16/0/H6 (NR_148601)	95.28
	966091*	86	x		Actinobacteria; Geodermatophilaceae	*Blastococcus colisei* BMG 822 (NR_149176)	99.33
	1093610	84	x		Firmicutes; Alicyclobacillaceae	*Tumebacillus luteolus* UC13 (NR_145584)	98.28
	166076	81	x		Actinobacteria; Rubrobacteraceae	*Rubrobacter spartanus* HPK2-2 (NR_158052)	93.10
	4402892	71	x		Alphaproteobacteria; Rhodospirillaceae	*Niveispirillum fermenti* CC-LY736 (NR_126263)	92.31
	453616*	71	x		Actinobacteria; Geodermatophilaceae	*Geodermatophilus pulveris* BMG 825 (NR_152017)	97.98
	237492	70	x		Alphaproteobacteria; yllobacteriaceae	*Chelativorans composti* Nis3 (NR_113183)	96.14
	704830	67	x		Actinobacteria; Geodermatophilaceae	*Geodermatophilus africanus* CF 11/1 (NR_108882)	98.43
	357423	57		x	Actinobacteria; Micromonosporaceae	*Krasilnikovia cinnamomea* 3-54(41) (NR_041337)	98.88
Endosphere	570692	123	x		Actinobacteria; Micromonosporaceae	*Phytohabitans flavus* K09-0627 (NR_113542)	98.88
	1110021	101	x		Actinobacteria; Geodermatophilaceae	*Modestobacter muralis* MDVD1 (NR_145554)	99.10
	251050	86	x		Epsilonproteobacteria; Helicobacteraceae	*Helicobacter brantae* MIT 04-9366 (NR_043799)	81.35
	1119093	81	x		Chloroflexi; Sphaerobacteraceae	*Sphaerobacter thermophilus* DSM 20745 (NR_074379)	85.65
	13074	76		x	Alphaproteobacteria; Sphingomonadaceae	*Sphingomonas agri* HKS-06 (NR_159163)	97.05
	844856	76	x		Firmicutes; Clostridiaceae	*Alkaliphilus metalliredigens* QYMF (NR_074633)	81.15
	1025949	71	x		Alphaproteobacteria; Phyllobacteriaceae	*Phyllobacterium myrsinacearum* NBRC 100019 (NR_113874)	99.32
	154591	70	x		Actinobacteria; Pseudonocardiaceae	*Actinophytocola oryzae* GMKU 367 (NR_116313)	98.65
	4465539*	68	x		Actinobacteria; Promicromonosporaceae	*Promicromonospora alba* 1C-HV12 (NR_148779)	99.33
	965129*	63	x		Alphaproteobacteria; Sphingomonadaceae	*Sphingomonas carotinifaciens* L9-754 (NR_159247)	99.09
	509487*	56	x		Actinobacteria; Pseudonocardiaceae	*Pseudonocardia hierapolitana* PM2084 (NR_126236)	99.33
	4371107	56	x		Actinobacteria; Conexibacteraceae	*Conexibacter stalactiti* YC2-25 (NR_157993)	98.49

*Relative abundance of OTU > 1% of all sequences.

## Discussion

### Composition and Diversity of Prokaryotic Communities Associated With Spineless Cacti: Differences Between Rhizosphere and Endosphere

Plant-associated microbes are recognized to have several beneficial effects on host plants, but they are affected by a wide variety of environmental and host-related factors, including compartment, host genotype and phenotype, geographic site, season, and soil chemistry. Our work consists in evaluating the composition of prokaryotic communities associated with the rhizosphere and endosphere of spineless *Opuntia ficus-indica.* Proteobacteria, Actinobacteria, Firmicutes, Chloroflexi, Acidobacteria, Bacteroidetes, Cyanobacteria, and Verrucomicrobia bacterial phyla dominated both the rhizosphere and endosphere of spineless cacti (Figure 1A). Actinobacteria, Bacteroidetes, Firmicutes, and Proteobacteria have been reported to be the dominating phyla in diverse plant-associated communities including *Arabidopsis* (Lundberg et al., 2012), *Populus* (Shakya et al., 2013), maize (Peiffer et al., 2013), grape (Zarraonaindia et al., 2015), rice (Edwards et al., 2015), and cacti and agave species (Desgarennes et al., 2014; Coleman-Derr et al., 2016; Fonseca-García et al., 2016, 2018). Furthermore, Acidobacteria has been identified as among the most represented phyla associated with the rhizosphere and the root endosphere of *M. geometrizans* and *O. robusta* (Fonseca-García et al., 2016). Moreover, Chloroflexi and Cyanobacteria were known by their multiple adaptations to environmental harshness such as soil biological crusts in arid environments (da Rocha et al., 2015; Maestre et al., 2015; Čapková et al., 2016; Zhang et al., 2016). Within the domain Archaea, these populations were not abundant in both the rhizosphere and endosphere of *Opuntia ficus-indica*. Findings similar to ours have been previously reported in plant-associated communities of agaves and cactus studies (Fonseca-García et al., 2016, 2018). However, different situations were also reported where Archaea were abundant such as in rice (Edwards et al., 2015) and olive (Müller et al., 2015). In this study, profiles of prokaryotic distribution associated with spineless *Opuntia ficus-indica* were clearly distinct between inside and outside roots (rhizosphere and endosphere samples), which is the major selective force shaping plant–microbe interactions in arid and semi-arid habitats. This is consistent with findings described for agaves and cacti (Desgarennes et al., 2014; Coleman-Derr et al., 2016; Fonseca-García et al., 2016, 2018).

### The Impact of the Aridity Gradient on Microbial Community Composition in Spineless Cacti

Under the background of climate change, the decrease in precipitation, the increase in temperature, and drought frequency in dryland environments are among the major factors influencing the composition of soil microbial communities, as well as yield and crop quality. In this study, we investigated the effect of the geographic location and the degree of aridity on the prokaryotic microbiome associated with spineless *Opuntia ficus-indica*. Hence, we analyzed the prokaryotic community structure from the rhizosphere and endosphere sampled from four locations across an aridity gradient including humid, semi-arid, upper-arid, and lower-arid (Figure 2). Our results revealed a significant variation of the prokaryotic community composition as a function of the aridity gradient and geographic location in both rhizosphere and endosphere samples. Alphaproteobacteria and Actinobacteria were significantly enriched with the increased degree of aridity in both the rhizosphere and endosphere. Betaproteobacteria and Verrucomicrobia phyla were only enriched in the endosphere and rhizosphere, respectively. However, Firmicutes and Betaproteobacteria decreased in the rhizosphere; Cyanobacteria and Deltaproteobacteria in endosphere. Thereby, we suggest that the prokaryotic community profiles in the rhizosphere and endosphere were altered considerably and differently according to the degree of aridity and the geographic location. Previous studies with diverse experiments evaluating the impact of drought stress on soil and root-associated microbiome revealed that Actinobacteria phyla, typically dominant in dryland environments, were significantly enriched in response to drought stress (Santos-Medellín et al., 2017; Ochoa-Hueso et al., 2018). Similar to our findings, Maestre et al. (2015) reported that Alphaproteobacteria increased with aridity.

The impact of the aridity gradient on spineless cactus-associated microbiome was also demonstrated at the OTU level where major OTUs detected in the rhizosphere and endosphere are as presented in Figure 3. Among these dominant OTUs, we identified that rhizosphere and endosphere increased the OTUs across the aridity gradient, which may play a significant role in stress and adaptation to aridity. Rhizosphere-increased OTUs were related to genera *Blastococcus*, *Geodermatophilus*, *Actinoplanes*, and *Promicromonospora* (Actinobacteria); endosphere-increased OTUs to genera *Promicromonospora*, *Pseudonocardia* (Actinobacteria), *Sphingomonas*, and *Agrobacterium* (Alphaproteobacteria class). *Sphingomonas*, *Burkholderia*, *Ralstonia*, *Bacillus*, *Geodermatophilus*, *Streptomyces*, *Amycolatopsis*, *Promicromonospora*, *Agrobacterium*, *Massilia*, *Pseudomonas*, and *Erwinia* are well-known as plant growth-promoting rhizobacteria or PGPR (Gopalakrishnan et al., 2015; Lamizadeh et al., 2016; Sathya et al., 2017; Gupta et al., 2018). Two rhizobacteria related to the genus *Bacillus* were isolated from Brazilian cacti, displaying plant growth-promotion abilities under drought conditions (Kavamura et al., 2013). *Sphingomonas*, *Burkholderia*, *Ralstonia*, *Bacillus*, *Rubrobacter*, *Blastococcus*, *Streptomyces*, *Amycolatopsis*, *Promicromonospora*, and *Pseudomonas* have been recognized as endophytic bacteria (Kandel et al., 2017; Singh and Dubey, 2018; Kuźniar et al., 2019). Endophytic bacteria and rhizoplane communities in cacti from the desert areas in Mexico have been identified by previous studies, revealing the effect of the association of bacteria, archaea, and fungi on seed germination and survival of cacti in their harsh natural environment (Puente et al., 2004a, b, 2009a,b; Fonseca-García et al., 2016).

### Factors Affecting the Assembly of Microbial Communities in Spineless Cacti Following the Aridity Gradient

We identified increased and decreased OTUs in inside and outside roots (rhizosphere and endosphere samples) across the aridity gradient by referring to their differential abundance (Supplementary Figure S3 and Supplementary Table S4). Decreased OTUs were affiliated to Actinobacteria, Acidobacteria, Firmicutes, Chloroflexi, Alphaproteobacteria (in both rhizosphere and endosphere samples), Verrucomicrobia, Bacteroidetes, Cyanobacteria (only in endosphere), and TM7 (only in the rhizosphere). However, increased OTUs belonged to Actinobacteria, Alphaproteobacteria, Betaproteobacteria, Bacteroidetes, Firmicutes and Chloroflexi (in both rhizosphere and endosphere), TM7, and Cyanobacteria (in endosphere). Dominant increased bacterial OTUs were related to Actinobacteria and Alphaproteobacteria. They were previously reported by other researchers studying the effect of drought stress on rice root-associated microbiome (Santos-Medellín et al., 2017). Our results indicated that microorganisms related to the increased bacterial OTUs could play an important role in the tolerance to aridity in the rhizosphere and endosphere associated with spineless cacti in the arid zone. These results suggest that microorganisms affected by aridity are sample type specific.

Co-occurrence correlation was performed firstly between increased and decreased OTUs and secondly between physicochemical soil parameters and increased and decreased OTUs according to the degree of aridity in both the rhizosphere and endosphere (Figure 4). A positive correlation between increased bacterial OTUs in the endosphere and rhizosphere was detected in co-occurrence patterns. However, potassium presents a negative correlation with them. Decreased bacterial OTUs were correlated positively with environmental variables such as iron, humidity, and phosphate, suggesting that they were affected by these physicochemical parameters. In water-limited soil, the decrease in ion contents including sodium, calcium carbonate, and potassium was reported (Bachar et al., 2010). In diverse experiments analyzing the influence of several factors on soil bacteria, chemical properties including pH and ion content have stressed an important role in describing community structure (Chodak et al., 2015; Gunnigle et al., 2017; Hartmann et al., 2017; Naylor and Coleman-Derr, 2018). Bacteria and nutrients are able to be transported more facilely via water which is limited in dry environments (Abu-Ashour et al., 1994). Dominant bacterial OTUs among all increased bacterial OTUs identified in the subnetwork of increased aridity degree in rhizosphere and endosphere samples are related to Actinobacteria and Alphaproteobacteria. These results suggest that these groups are able to tolerate drought conditions. Some bacterial species related to the Actinobacteria phylum are able to form spores to resist to desiccation and survive under drought conditions (Singh et al., 2007). Several studies have reported that the enrichment of Alphaproteobacteria and Actinobacteria in the rhizosphere where carbon is available was increased due to root exudates (Smalla et al., 2001; Fierer et al., 2007). PhyloChip hybridization analysis revealed that Alphaproteobacteria and Actinobacteria were more abundant in semi-arid deserts (Ding et al., 2013). Bacterial species isolated from the rhizosphere of three cactus plant species (*Mammillaria carnea*, *Opuntia pilifera*, and *Stenocereus stellatus*) were mainly affiliated with Alphaproteobacteria, Actinobacteria, and Firmicutes (Aguirre-Garrido et al., 2012). Torres-Cortés et al. (2012) demonstrated that Actinobacteria was the most dominant phyla in the rhizosphere of the cactus species *Mammillaria carnea* during the dry season using the pyrosequencing method. Our findings displayed that bacterial species related to *Blastococcus*, *Geodermatophilus*, *Pseudonocardia*, *Promicromonospora*, and *Sphingomonas* genera formed five dominant hubs. *Blastococcus* and *Geodermatophilus* were increased especially in the rhizosphere whereas *Pseudonocardia*, *Promicromonospora*, and *Sphingomonas* were more abundant in the endosphere across the aridity gradient. These hubs could play a principal role in the arid zones associated with spineless cacti. Indeed, several studies reported that these strains were often associated with deserts and biocrust habitats and their adaptation to extreme environmental conditions, such as high salt concentration, low relative humidity, and high UV radiation, has been described (Hussain et al., 2019; Pombubpa et al., 2019). Moreover, bacterial species related to these genera have been defined as endophytes associated with arid plants and some of them possess the ability to protect the host plants and promote their growth (Trujillo et al., 2015; Singh and Dubey, 2018). These endophytic bacteria may play crucial roles in maintenance and sustenance of specific arid habitats.

## Conclusion

In the present work, we used a next-generation sequencing approach to assess bacterial and archaeal diversity and composition in rhizosphere and endosphere samples associated with *Opuntia ficus-indica* spineless cacti along an increased aridity gradient in Tunisia. Our findings revealed that microbial composition and abundance were linked to the rhizosphere and endosphere. Interestingly, Actinobacteria, Firmicutes, and Chloroflexi phyla were more abundant in the rhizosphere and inversely Cyanobacteria, Proteobacteria (especially Gammaproteobacteria and Betaproteobacteria classes), and TM7 phyla were more dominant in the endosphere. Along the climatic aridity gradient, Alphaproteobacteria and Actinobacteria were significantly increased in both rhizosphere and endosphere. However, Betaproteobacteria and Verrucomicrobia were enriched in the endosphere and rhizosphere, respectively. Similarly, Firmicutes and Betaproteobacteria decreased in the rhizosphere; Cyanobacteria and Deltaproteobacteria in the endosphere. Indeed, specific OTUs were detected in each bioclimatic zone. These results suggest that the aridity gradient potentially shaped the diversity and composition of bacteria and archaea in the rhizosphere and endosphere associated with spineless cactus. Interestingly, in rhizosphere and endosphere samples increased and decreased OTUs along the aridity gradient were identified and their co-occurrence in correlation with soil parameters was investigated. Five hubs related to *Blastococcus* and *Geodermatophilus* (in rhizosphere) and to *Pseudonocardia*, *Promicromonospora*, and *Sphingomonas* (in endosphere) may be considered as indicators of aridity. Thus, we predict that the climatic aridity will shift significantly the cactus microbial diversity in the rhizosphere and endosphere and occurring taxa being specialized to this zone. The effect of other abiotic (salinity, elevation, spatial…) and biotic (diverse species) parameters on the bacterial and fungal microbiome associated with cacti will be monitored.

## Data Availability Statement

The datasets generated for this study can be found in the Bacterial sequence data of the DGGE bands has been submitted to GenBank and the assigned accession numbers were from MK208464 to MK208475. 16S Raw reads have been deposited in the Short Read Archive of NCBI under project no. PRJNA511384.

## Author Contributions

FK, MG, AM, and SS conceived and designed the study. AC coordinated the sampling and performed the soil physicochemical analyses. NM performed the molecular analyses. FK and MG performed the bioinformatic and statistical analyses. FK wrote the original draft of the manuscript. All authors read and contributed to the review and editing of the final manuscript.

## Conflict of Interest

The authors declare that the research was conducted in the absence of any commercial or financial relationships that could be construed as a potential conflict of interest.

## Footnotes

1 http://nmf.r-forge.r-project.org/aheatmap.html
