# Supplementary material for: Climatic Aridity Gradient Modulates the Diversity of the Rhizosphere and Endosphere Bacterial Microbiomes of Opuntia ficus-indica
Source: Front Microbiol. 2020 Jul 28;11:1622. doi: 10.3389/fmicb.2020.01622 (PMC7401614; doi:10.3389/fmicb.2020.01622)
Supplement: Supplementary file 1 [file Data_Sheet_1.pdf]

Table S1: HSD-Tukey test for differences between physico-chemical parameters across the different bioclimatic zones.

Multiple Comparisons

Tukey HSD

Dependent Variable	(I) Stage	(J) Stage	Mean Difference (I-J)	Std. Error	Sig.	95% Confidence Interval	
						Lower Bound	Upper Bound
**pH**	Upper_Arid	Lower-Arid	-1.037	0.441	0.125	-2.29	0.22
		Semi-Arid	-0.117	0.441	0.993	-1.37	1.14
		Humid	-0.351	0.441	0.855	-1.6	0.9
	Lower-Arid	Upper_Arid	1.037	0.441	0.125	-0.22	2.29
		Semi-Arid	0.92	0.557	0.379	-0.66	2.5
		Humid	0.686	0.557	0.617	-0.9	2.27
	Semi-Arid	Upper_Arid	0.117	0.441	0.993	-1.14	1.37
		Lower-Arid	-0.92	0.557	0.379	-2.5	0.66
		Humid	-0.234	0.557	0.974	-1.82	1.35
	Humid	Upper_Arid	0.351	0.441	0.855	-0.9	1.6
		Lower-Arid	-0.686	0.557	0.617	-2.27	0.9
		Semi-Arid	0.234	0.557	0.974	-1.35	1.82
**Humidity**	Upper_Arid	Lower-Arid	-0.643	1.423	**0.038**	-4.69	3.4
		Semi-Arid	2.586	1.423	**0.03**	-1.46	6.63
		Humid	0.087	1.423	**0.04**	-3.96	4.13
	Lower-Arid	Upper_Arid	0.643	1.423	**0.038**	-3.4	4.69
		Semi-Arid	3.229	1.8	**0.03**	-1.89	8.35
		Humid	0.73	1.8	**0.027**	-4.39	5.85
	Semi-Arid	Upper_Arid	-2.586	1.423	**0.03**	-6.63	1.46
		Lower-Arid	-3.229	1.8	**0.03**	-8.35	1.89
		Humid	-2.499	1.8	**0.023**	-7.62	2.62

	Humid	Upper_Arid	-0.087	1.423	**0.04**	-4.13	3.96
		Lower-Arid	-0.73	1.8	**0.027**	-5.85	4.39
		Semi-Arid	2.499	1.8	**0.023**	-2.62	7.62
**TP**	Upper_Arid	Lower-Arid	-6.921	3.386	**0.021**	-16.54	2.7
		Semi-Arid	-8.518	3.386	**0.033**	-18.14	1.11
		Humid	-10.534	3.386	**0.029**	-20.16	-0.91
	Lower-Arid	Upper_Arid	6.921	3.386	**0.021**	-2.7	16.54
		Semi-Arid	-1.597	4.282	**0.048**	-13.77	10.58
		Humid	-3.613	4.282	**0.043**	-15.79	8.56
	Semi-Arid	Upper_Arid	8.518	3.386	**0.033**	-1.11	18.14
		Lower-Arid	1.597	4.282	**0.048**	-10.58	13.77
		Humid	-2.016	4.282	**0.046**	-14.19	10.16
	Humid	Upper_Arid	10.534	3.386	**0.029**	-0.91	20.16
		Lower-Arid	3.613	4.282	**0.043**	-8.56	15.79
		Semi-Arid	2.016	4.282	**0.046**	-10.16	14.19
**TOC**	Upper_Arid	Lower-Arid	-2.009	0.444	**0.002**	-3.27	-0.75
		Semi-Arid	0.207	0.444	**0.036**	-1.05	1.47
		Humid	-3.489	0.444	**0**	-4.75	-2.23
	Lower-Arid	Upper_Arid	2.009	0.444	**0.002**	0.75	3.27
		Semi-Arid	2.216	0.561	**0.005**	0.62	3.81
		Humid	-1.479	0.561	**0.044**	-3.08	0.12
	Semi-Arid	Upper_Arid	-0.207	0.444	**0.036**	-1.47	1.05
		Lower-Arid	-2.216	0.561	**0.005**	-3.81	-0.62
		Humid	-3.696	0.561	**0**	-5.29	-2.1
	Humid	Upper_Arid	3.489	0.444	**0**	2.23	4.75
		Lower-Arid	1.479	0.561	**0.044**	-0.12	3.08
		Semi-Arid	3.696	0.561	**0**	2.1	5.29
**TN**	Upper_Arid	Lower-Arid	0.19	0.2	0.779	-0.38	0.76
		Semi-Arid	0.135	0.2	0.905	-0.43	0.7
		Humid	0.282	0.2	0.51	-0.29	0.85

	Lower-Arid	Upper_Arid	-0.19	0.2	0.779	-0.76	0.38
		Semi-Arid	-0.054	0.253	0.996	-0.77	0.66
		Humid	0.092	0.253	0.983	-0.63	0.81
	Semi-Arid	Upper_Arid	-0.135	0.2	0.905	-0.7	0.43
		Lower-Arid	0.054	0.253	0.996	-0.66	0.77
		Humid	0.147	0.253	0.937	-0.57	0.87
	Humid	Upper_Arid	-0.282	0.2	0.51	-0.85	0.29
		Lower-Arid	-0.092	0.253	0.983	-0.81	0.63
		Semi-Arid	-0.147	0.253	0.937	-0.87	0.57
**Fe^{3+}**	Upper_Arid	Lower-Arid	-3.734	0.789	**0.001**	-5.98	-1.49
		Semi-Arid	-4.442	0.789	**0**	-6.69	-2.2
		Humid	-7.201	0.789	**0**	-9.44	-4.96
	Lower-Arid	Upper_Arid	3.734	0.789	**0.001**	1.49	5.98
		Semi-Arid	-0.708	0.998	**0.048**	-3.55	2.13
		Humid	-3.467	0.998	**0.014**	-6.3	-0.63
	Semi-Arid	Upper_Arid	4.442	0.789	**0**	2.2	6.69
		Lower-Arid	0.708	0.998	**0.048**	-2.13	3.55
		Humid	-2.759	0.998	**0.049**	-5.6	0.08
	Humid	Upper_Arid	7.201	0.789	**0**	4.96	9.44
		Lower-Arid	3.467	0.998	**0.014**	0.63	6.3
		Semi-Arid	2.759	0.998	**0.049**	-0.08	5.6
**Ca^{2+}**	Upper_Arid	Lower-Arid	-2253.354	800.591	**0.043**	-4529.08	22.37
		Semi-Arid	1648.66	800.591	**0.02**	-627.07	3924.39
		Humid	-1833.007	800.591	**0.014**	-4108.74	442.72
	Lower-Arid	Upper_Arid	2253.354	800.591	**0.043**	-22.37	4529.08
		Semi-Arid	3902.014	1012.677	**0.006**	1023.42	6780.61
		Humid	420.347	1012.677	**0.037**	-2458.25	3298.94
	Semi-Arid	Upper_Arid	-1648.66	800.591	**0.02**	-3924.39	627.07
		Lower-Arid	-3902.014	1012.677	**0.006**	-6780.61	-1023.42

		Humid	-3481.667	1012.677	**0.015**	-6360.26	-603.07
	Humid	Upper_Arid	1833.007	800.591	**0.014**	-442.72	4108.74
		Lower-Arid	-420.347	1012.677	**0.037**	-3298.94	2458.25
		Semi-Arid	3481.667	1012.677	**0.015**	603.07	6360.26
**K^+**	Upper_Arid	Lower-Arid	-80.343	29.827	**0.037**	-165.13	4.44
		Semi-Arid	0.742	29.827	**0.01**	-84.04	85.53
		Humid	19.68	29.827	**0.031**	-65.1	104.46
	Lower-Arid	Upper_Arid	80.343	29.827	**0.037**	-4.44	165.13
		Semi-Arid	81.085	37.728	**0.017**	-26.16	188.33
		Humid	100.022	37.728	**0.042**	-7.22	207.27
	Semi-Arid	Upper_Arid	-0.742	29.827	**0.01**	-85.53	84.04
		Lower-Arid	-81.085	37.728	**0.017**	-188.33	26.16
		Humid	18.938	37.728	**0.035**	-88.31	126.18
	Humid	Upper_Arid	-19.68	29.827	**0.031**	-104.46	65.1
		Lower-Arid	-100.022	37.728	**0.042**	-207.27	7.22
		Semi-Arid	-18.938	37.728	**0.035**	-126.18	88.31
**CEC**	Upper_Arid	Lower-Arid	-2.058	2.895	**0.048**	-10.29	6.17
		Semi-Arid	3.458	2.895	**0.036**	-4.77	11.69
		Humid	-12.775	2.895	**0.002**	-21.01	-4.54
	Lower-Arid	Upper_Arid	2.058	2.895	**0.048**	-6.17	10.29
		Semi-Arid	5.517	3.662	**0.045**	-4.89	15.93
		Humid	-10.717	3.662	**0.042**	-21.13	-0.31
	Semi-Arid	Upper_Arid	-3.458	2.895	**0.036**	-11.69	4.77
		Lower-Arid	-5.517	3.662	**0.045**	-15.93	4.89
		Humid	-16.233	3.662	**0.002**	-26.64	-5.82
	Humid	Upper_Arid	12.775	2.895	**0.002**	4.54	21.01
		Lower-Arid	10.717	3.662	**0.042**	0.31	21.13
		Semi-Arid	16.233	3.662	**0.002**	5.82	26.64

Table S2: Blast results on bacterial 16S rRNA sequences derived from excised DGGE bands from rhizosphere and endosphere samples of the spineless *Optunia ficus indica* from each bioclimatic zone.

	Band #	Length (bp)	Phylogenetic affiliation	Best match/ accession number	% of similarity	Source
**Rhizosphere**	*BS4*	*149*	Actinobacteria. Rubrobacteraceae	*Rubrobacter xylanophilus* DSM 9941 / (NR_074552)	***95***	Thermally polluted effluent
	BS5	*119*	Firmicutes. Planococcaceae	Bacillus beijingensis strain ge10/ NR_044192	***86***	ginseng root
	BS7	*152*	Firmicutes. Bacillaceae	*Bacillus koreensis* BR030 / (NR_043084)	***99***	Rhizosphere of willowroots in Korea
	BS8	*146*	Actinobacteria. Micrococcaceae	*Arthrobacter bambusae* THG-GM18 / (NR_133968)	***91***	Soil of a bamboogrove
	BS9	*98*	Gammaproteobacteria. Colwelliaceae	Uncultured Colwellia sp. clone P04/ KT336095	***77***	manganese nodule
	BS12	*169*	Firmicutes. Bacillaceae	*Bacillus huizhouensis* GSS03 / (NR_133974)	***98***	Paddy field soil
	BS15	*137*	Actinobacteria. Geodermatophilaceae	*Geodermatophilus brasiliensis* Tu6233 / (NR_126197)	***97***	Brazilian soil
	BS16	*116*	Deltaproteobacteria. Geobacteraceae	*Geobacter sulfurreducens subsp. ethanolicus* OSK2A / (NR_132673)	***81***	lotus field mud
	BS18	*139*	Actinobacteria. Frankiaceae	*Frankia inefficax* EuI1c/ (KX695197)	***99***	Root nodules of *Elaeagnusumbellata*
**Endosphere**	*RB1*	*105*	Bacteria. environmental samples	Uncultured bacterium clone HC1_3658/ (KP706269)	***82***	White microbial mat from Hopkins Chocolate Cave. Lava Beds National Monument
	RB4	*112*	Bacteria. environmental samples	Uncultured bacterium clone Otu01917/ (KX996879)	***86***	
	RB7	*149*	Betaproteobacteria. Oxalobacteraceae	*Oxalicibacterium horti* OD1 / (NR_112833)	***94***	Garden soil
	RB11	*147*	Bacteria. environmental samples	Uncultured bacterium isolate DGGE gel band F19/ (KC608042)	***80***	Eucalyptus wood chips
	RB13	*97*	Gammaproteobacteria. Enterobacteriaceae	*Citrobacter freundii* UrCAN5/(JX133227)	***92***	rhizospheric soil
	RB22	*174*	Alphaproteobacteria. Rhizobiaceae	*Agrobacterium tumefaciens* isolate AG-RST/(LT718194)	***73***	Guava roots
	RB24	*122*	Bacteria	Uncultured bacterium clone GB7N87002D2TTD/ (HM674756)	***78***	unvegetated soil environments on Signy Island
	RB29	*130*	Actinobacteria. Streptomycetaceae	*Streptomyces fabae* T66 / (NR_145617)	***98***	forest soil
	RB30	*137*	Actinobacteria. Pseudonocardiaceae	*Amycolatopsis roodepoortensis* M29/ (NR_134695)	***97***	South African soils

Table S3: HSD-Tukey test for differences between phylum/class across the different bioclimatic zones.

Rhizosphere

Multiple Comparisons

Tukey HSD

Dependent Variable	(I) Phylum	(J) Phylum	Mean Difference (I-J)	Std. Error	Sig.	95% Confidence Interval	
						Lower Bound	Upper Bound
**Euryarchaeota**	Upper_Arid	Lower_Arid	0.584	0.762	0.994	-1.87	3.04
		Semi_Arid	0.312	0.762	1	-2.15	2.77
		Humid	0.642	0.762	0.989	-1.82	3.1
	Lower_Arid	Upper_Arid	-0.584	0.762	0.994	-3.04	1.87
		Semi_Arid	-0.272	0.964	1	-3.38	2.84
		Humid	0.058	0.964	1	-3.05	3.17
	Semi_Arid	Upper_Arid	-0.312	0.762	1	-2.77	2.15
		Lower_Arid	0.272	0.964	1	-2.84	3.38
		Humid	0.331	0.964	1	-2.78	3.44
	Humid	Upper_Arid	-0.642	0.762	0.989	-3.1	1.82
		Lower_Arid	-0.058	0.964	1	-3.17	3.05
		Semi_Arid	-0.331	0.964	1	-3.44	2.78
**Acidobacteria**	Upper_Arid	Lower_Arid	-0.199	1.008	1	-3.45	3.05
		Semi_Arid	-2.307	1.008	0.328	-5.56	0.95
		Humid	0.008	1.008	1	-3.24	3.26
	Lower_Arid	Upper_Arid	0.199	1.008	1	-3.05	3.45
		Semi_Arid	-2.108	1.275	0.716	-6.22	2.01
		Humid	0.208	1.275	1	-3.91	4.32
	Semi_Arid	Upper_Arid	2.307	1.008	0.328	-0.95	5.56
		Lower_Arid	2.108	1.275	0.716	-2.01	6.22
		Humid	2.316	1.275	0.614	-1.8	6.43
	Humid	Upper_Arid	-0.008	1.008	1	-3.26	3.24

		Lower_Arid	-0.208	1.275	1	-4.32	3.91
		Semi_Arid	-2.316	1.275	0.614	-6.43	1.8
**Actinobacteria**	Upper_Arid	Lower_Arid	10.891	5.465	**0.04**	-6.74	28.53
		Semi_Arid	14.004	5.465	**0.02**	-3.63	31.64
		Humid	30.55	5.465	**0**	12.92	48.18
	Lower_Arid	Upper_Arid	-10.891	5.465	**0.04**	-28.53	6.74
		Semi_Arid	3.113	6.913	**0.04**	-19.19	25.42
		Humid	19.659	6.913	**0.011**	-2.65	41.97
	Semi_Arid	Upper_Arid	-14.004	5.465	**0.02**	-31.64	3.63
		Lower_Arid	-3.113	6.913	**0.04**	-25.42	19.19
		Humid	16.546	6.913	**0.027**	-5.76	38.85
	Humid	Upper_Arid	-30.55	5.465	**0**	-48.18	-12.92
		Lower_Arid	-19.659	6.913	**0.011**	-41.97	2.65
		Semi_Arid	-16.546	6.913	**0.027**	-38.85	5.76
**Armatimonadetes**	Upper_Arid	Lower_Arid	-0.003	0.013	1	-0.04	0.04
		Semi_Arid	-0.044	0.013	**0.033**	-0.08	0
		Humid	0.001	0.013	1	-0.04	0.04
	Lower_Arid	Upper_Arid	0.003	0.013	1	-0.04	0.04
		Semi_Arid	-0.04	0.016	0.239	-0.09	0.01
		Humid	0.004	0.016	1	-0.05	0.06
	Semi_Arid	Upper_Arid	0.044	0.013	**0.033**	0	0.08
		Lower_Arid	0.04	0.016	0.239	-0.01	0.09
		Humid	0.045	0.016	0.141	-0.01	0.1
	Humid	Upper_Arid	-0.001	0.013	1	-0.04	0.04
		Lower_Arid	-0.004	0.016	1	-0.06	0.05
		Semi_Arid	-0.045	0.016	0.141	-0.1	0.01
**Bacteroidetes**	Upper_Arid	Lower_Arid	-0.697	0.901	0.993	-3.6	2.21
		Semi_Arid	-0.75	0.901	0.99	-3.66	2.16
		Humid	0.295	0.901	1	-2.61	3.2
	Lower_Arid	Upper_Arid	0.697	0.901	0.993	-2.21	3.6

		Semi_Arid	-0.054	1.139	1	-3.73	3.62
		Humid	0.992	1.139	0.987	-2.68	4.67
	Semi_Arid	Upper_Arid	0.75	0.901	0.99	-2.16	3.66
		Lower_Arid	0.054	1.139	1	-3.62	3.73
		Humid	1.045	1.139	0.982	-2.63	4.72
	Humid	Upper_Arid	-0.295	0.901	1	-3.2	2.61
		Lower_Arid	-0.992	1.139	0.987	-4.67	2.68
		Semi_Arid	-1.045	1.139	0.982	-4.72	2.63
**Chloroflexi**	Upper_Arid	Lower_Arid	-0.659	1.668	1	-6.04	4.72
		Semi_Arid	1.016	1.668	0.999	-4.37	6.4
		Humid	3.291	1.668	0.513	-2.09	8.67
	Lower_Arid	Upper_Arid	0.659	1.668	1	-4.72	6.04
		Semi_Arid	1.675	2.11	0.992	-5.13	8.48
		Humid	3.951	2.11	0.578	-2.86	10.76
	Semi_Arid	Upper_Arid	-1.016	1.668	0.999	-6.4	4.37
		Lower_Arid	-1.675	2.11	0.992	-8.48	5.13
		Humid	2.276	2.11	0.957	-4.53	9.08
	Humid	Upper_Arid	-3.291	1.668	0.513	-8.67	2.09
		Lower_Arid	-3.951	2.11	0.578	-10.76	2.86
		Semi_Arid	-2.276	2.11	0.957	-9.08	4.53
**Cyanobacteria**	Upper_Arid	Lower_Arid	0.269	5.512	1	-17.52	18.06
		Semi_Arid	0.245	5.512	1	-17.54	18.03
		Humid	0.191	5.512	1	-17.6	17.98
	Lower_Arid	Upper_Arid	-0.269	5.512	1	-18.06	17.52
		Semi_Arid	-0.025	6.973	1	-22.52	22.47
		Humid	-0.078	6.973	1	-22.58	22.42
	Semi_Arid	Upper_Arid	-0.245	5.512	1	-18.03	17.54
		Lower_Arid	0.025	6.973	1	-22.47	22.52
		Humid	-0.054	6.973	1	-22.55	22.44
	Humid	Upper_Arid	-0.191	5.512	1	-17.98	17.6

		Lower_Arid	0.078	6.973	1	-22.42	22.58
		Semi_Arid	0.054	6.973	1	-22.44	22.55
**Firmicutes**	Upper_Arid	Lower_Arid	12.526	3.831	**0.045**	0.17	24.89
		Semi_Arid	-2.883	3.831	0.994	-15.24	9.48
		Humid	-18.954	3.831	**0**	-31.31	-6.59
	Lower_Arid	Upper_Arid	-12.526	3.831	**0.045**	-24.89	-0.17
		Semi_Arid	-15.409	4.846	0.056	-31.04	0.23
		Humid	-31.48	4.846	**0**	-47.11	-15.84
	Semi_Arid	Upper_Arid	2.883	3.831	0.994	-9.48	15.24
		Lower_Arid	15.409	4.846	0.056	-0.23	31.04
		Humid	-16.07	4.846	**0.04**	-31.71	-0.44
	Humid	Upper_Arid	18.954	3.831	**0**	6.59	31.31
		Lower_Arid	31.48	4.846	**0**	15.84	47.11
		Semi_Arid	16.07	4.846	**0.04**	0.44	31.71
**Gemmatimonadetes**	Upper_Arid	Lower_Arid	-0.045	0.046	0.973	-0.19	0.1
		Semi_Arid	-0.378	0.046	**0**	-0.53	-0.23
		Humid	-2.064	0.046	**0**	-2.21	-1.92
	Lower_Arid	Upper_Arid	0.045	0.046	0.973	-0.1	0.19
		Semi_Arid	-0.333	0.057	**0**	-0.52	-0.15
		Humid	-2.019	0.057	**0**	-2.2	-1.83
	Semi_Arid	Upper_Arid	0.378	0.046	**0**	0.23	0.53
		Lower_Arid	0.333	0.057	**0**	0.15	0.52
		Humid	-1.686	0.057	**0**	-1.87	-1.5
	Humid	Upper_Arid	2.064	0.046	**0**	1.92	2.21
		Lower_Arid	2.019	0.057	**0**	1.83	2.2
		Semi_Arid	1.686	0.057	**0**	1.5	1.87
**Nitrospirae**	Upper_Arid	Lower_Arid	-0.004	0.03	1	-0.1	0.09
		Semi_Arid	-0.304	0.03	**0**	-0.4	-0.21
		Humid	0	0.03	1	-0.1	0.1
	Lower_Arid	Upper_Arid	0.004	0.03	1	-0.09	0.1

		Semi_Arid	-0.299	0.038	**0**	-0.42	-0.18
		Humid	0.004	0.038	1	-0.12	0.13
	Semi_Arid	Upper_Arid	0.304	0.03	**0**	0.21	0.4
		Lower_Arid	0.299	0.038	**0**	0.18	0.42
		Humid	0.304	0.038	**0**	0.18	0.43
	Humid	Upper_Arid	0	0.03	1	-0.1	0.1
		Lower_Arid	-0.004	0.038	1	-0.13	0.12
		Semi_Arid	-0.304	0.038	**0**	-0.43	-0.18
**Alphaproteobacteria**	Upper_Arid	Lower_Arid	-15.395	3.519	**0.002**	-26.75	-4.04
		Semi_Arid	-5.81	3.519	**0.017**	-17.17	5.54
		Humid	-0.774	3.519	**0.04**	-12.13	10.58
	Lower_Arid	Upper_Arid	15.395	3.519	**0.002**	4.04	26.75
		Semi_Arid	9.585	4.451	**0.04**	-4.78	23.95
		Humid	14.621	4.451	**0.044**	0.26	28.98
	Semi_Arid	Upper_Arid	5.81	3.519	**0.017**	-5.54	17.17
		Lower_Arid	-9.585	4.451	**0.04**	-23.95	4.78
		Humid	5.036	4.451	**0.045**	-9.33	19.4
	Humid	Upper_Arid	0.774	3.519	**0.04**	-10.58	12.13
		Lower_Arid	-14.621	4.451	**0.044**	-28.98	-0.26
		Semi_Arid	-5.036	4.451	**0.045**	-19.4	9.33
**Betaproteobacteria**	Upper_Arid	Lower_Arid	-6.516	5.381	**0.023**	-23.88	10.85
		Semi_Arid	-1.9	5.381	**0.04**	-19.26	15.46
		Humid	-14.256	5.381	**0.017**	-31.62	3.11
	Lower_Arid	Upper_Arid	6.516	5.381	**0.023**	-10.85	23.88
		Semi_Arid	4.616	6.806	**0.039**	-17.35	26.58
		Humid	-7.74	6.806	**0.044**	-29.7	14.22
	Semi_Arid	Upper_Arid	1.9	5.381	**0.04**	-15.46	19.26
		Lower_Arid	-4.616	6.806	**0.039**	-26.58	17.35
		Humid	-12.357	6.806	**0.014**	-34.32	9.61
	Humid	Upper_Arid	14.256	5.381	**0.017**	-3.11	31.62

		Lower_Arid	7.74	6.806	**0.044**	-14.22	29.7
		Semi_Arid	12.357	6.806	**0.014**	-9.61	34.32
**Deltaproteobacteria**	Upper_Arid	Lower_Arid	-0.48	0.168	0.115	-1.02	0.06
		Semi_Arid	-0.73	0.168	**0.003**	-1.27	-0.19
		Humid	-0.366	0.168	0.39	-0.91	0.18
	Lower_Arid	Upper_Arid	0.48	0.168	0.115	-0.06	1.02
		Semi_Arid	-0.25	0.213	0.934	-0.94	0.44
		Humid	0.114	0.213	0.999	-0.57	0.8
	Semi_Arid	Upper_Arid	0.73	0.168	**0.003**	0.19	1.27
		Lower_Arid	0.25	0.213	0.934	-0.44	0.94
		Humid	0.364	0.213	0.681	-0.32	1.05
	Humid	Upper_Arid	0.366	0.168	0.39	-0.18	0.91
		Lower_Arid	-0.114	0.213	0.999	-0.8	0.57
		Semi_Arid	-0.364	0.213	0.681	-1.05	0.32
**Gammaproteobacteria**	Upper_Arid	Lower_Arid	0.384	6.684	1	-21.18	21.95
		Semi_Arid	0.165	6.684	1	-21.4	21.73
		Humid	0.878	6.684	1	-20.69	22.45
	Lower_Arid	Upper_Arid	-0.384	6.684	1	-21.95	21.18
		Semi_Arid	-0.219	8.455	1	-27.5	27.06
		Humid	0.494	8.455	1	-26.79	27.77
	Semi_Arid	Upper_Arid	-0.165	6.684	1	-21.73	21.4
		Lower_Arid	0.219	8.455	1	-27.06	27.5
		Humid	0.712	8.455	1	-26.57	27.99
	Humid	Upper_Arid	-0.878	6.684	1	-22.45	20.69
		Lower_Arid	-0.494	8.455	1	-27.77	26.79
		Semi_Arid	-0.712	8.455	1	-27.99	26.57
**Tenericutes**	Upper_Arid	Lower_Arid	0.001	0.01	1	-0.03	0.03
		Semi_Arid	-0.024	0.01	0.31	-0.06	0.01
		Humid	0.003	0.01	1	-0.03	0.04
	Lower_Arid	Upper_Arid	-0.001	0.01	1	-0.03	0.03

		Semi_Arid	-0.025	0.013	0.573	-0.07	0.02
		Humid	0.002	0.013	1	-0.04	0.04
	Semi_Arid	Upper_Arid	0.024	0.01	0.31	-0.01	0.06
		Lower_Arid	0.025	0.013	0.573	-0.02	0.07
		Humid	0.027	0.013	0.465	-0.02	0.07
	Humid	Upper_Arid	-0.003	0.01	1	-0.04	0.03
		Lower_Arid	-0.002	0.013	1	-0.04	0.04
		Semi_Arid	-0.027	0.013	0.465	-0.07	0.02
**TM7**	Upper_Arid	Lower_Arid	-0.143	0.517	1	-1.81	1.52
		Semi_Arid	-0.085	0.517	1	-1.75	1.58
		Humid	-0.007	0.517	1	-1.67	1.66
	Lower_Arid	Upper_Arid	0.143	0.517	1	-1.52	1.81
		Semi_Arid	0.058	0.653	1	-2.05	2.17
		Humid	0.136	0.653	1	-1.97	2.24
	Semi_Arid	Upper_Arid	0.085	0.517	1	-1.58	1.75
		Lower_Arid	-0.058	0.653	1	-2.17	2.05
		Humid	0.078	0.653	1	-2.03	2.19
	Humid	Upper_Arid	0.007	0.517	1	-1.66	1.67
		Lower_Arid	-0.136	0.653	1	-2.24	1.97
		Semi_Arid	-0.078	0.653	1	-2.19	2.03
**Verrucomicrobia**	Upper_Arid	Lower_Arid	-0.654	0.215	**0.036**	-1.35	0.04
		Semi_Arid	-0.936	0.215	**0.003**	-1.63	-0.24
		Humid	0.105	0.215	**0.04**	-0.59	0.8
	Lower_Arid	Upper_Arid	0.654	0.215	**0.036**	-0.04	1.35
		Semi_Arid	-0.281	0.272	**0.036**	-1.16	0.6
		Humid	0.759	0.272	**0.013**	-0.12	1.64
	Semi_Arid	Upper_Arid	0.936	0.215	**0.003**	0.24	1.63
		Lower_Arid	0.281	0.272	**0.036**	-0.6	1.16
		Humid	1.041	0.272	**0.011**	0.16	1.92
	Humid	Upper_Arid	-0.105	0.215	**0.04**	-0.8	0.59

		Lower_Arid	-0.759	0.272	**0.013**	-1.64	0.12
		Semi_Arid	-1.041	0.272	**0.011**	-1.92	-0.16
**Unassigned**	Upper_Arid	Lower_Arid	0.133	0.185	0.996	-0.47	0.73
		Semi_Arid	0.401	0.185	0.398	-0.2	1
		Humid	0.448	0.185	0.266	-0.15	1.05
	Lower_Arid	Upper_Arid	-0.133	0.185	0.996	-0.73	0.47
		Semi_Arid	0.268	0.234	0.942	-0.49	1.02
		Humid	0.315	0.234	0.875	-0.44	1.07
	Semi_Arid	Upper_Arid	-0.401	0.185	0.398	-1	0.2
		Lower_Arid	-0.268	0.234	0.942	-1.02	0.49
		Humid	0.047	0.234	1	-0.71	0.8
	Humid	Upper_Arid	-0.448	0.185	0.266	-1.05	0.15
		Lower_Arid	-0.315	0.234	0.875	-1.07	0.44
		Semi_Arid	-0.047	0.234	1	-0.8	0.71

Endosphere

Multiple Comparisons

Tukey HSD

Dependent Variable	(I) Phylum	(J) Phylum	Mean Difference (I-J)	Std. Error	Sig.	95% Confidence Interval	
						Lower Bound	Upper Bound
**Euryarchaeota**	Upper_Arid	Lower_Arid	0.021	0.762	1	-2.44	2.48
		Semi_Arid	0.061	0.762	1	-2.4	2.52
		Humid	0.059	0.762	1	-2.4	2.52
	Lower_Arid	Upper_Arid	-0.021	0.762	1	-2.48	2.44
		Semi_Arid	0.04	0.964	1	-3.07	3.15
		Humid	0.038	0.964	1	-3.07	3.15
	Semi_Arid	Upper_Arid	-0.061	0.762	1	-2.52	2.4
		Lower_Arid	-0.04	0.964	1	-3.15	3.07
		Humid	-0.002	0.964	1	-3.11	3.11
	Humid	Upper_Arid	-0.059	0.762	1	-2.52	2.4
		Lower_Arid	-0.038	0.964	1	-3.15	3.07
		Semi_Arid	0.002	0.964	1	-3.11	3.11
**Acidobacteria**	Upper_Arid	Lower_Arid	1.296	1.008	0.898	-1.96	4.55
		Semi_Arid	0.045	1.008	1	-3.21	3.3
		Humid	0.818	1.008	0.991	-2.43	4.07
	Lower_Arid	Upper_Arid	-1.296	1.008	0.898	-4.55	1.96
		Semi_Arid	-1.251	1.275	0.974	-5.36	2.86
		Humid	-0.478	1.275	1	-4.59	3.64
	Semi_Arid	Upper_Arid	-0.045	1.008	1	-3.3	3.21
		Lower_Arid	1.251	1.275	0.974	-2.86	5.36
		Humid	0.773	1.275	0.999	-3.34	4.89
	Humid	Upper_Arid	-0.818	1.008	0.991	-4.07	2.43
		Lower_Arid	0.478	1.275	1	-3.64	4.59

		Semi_Arid	-0.773	1.275	0.999	-4.89	3.34
**Actinobacteria**	Upper_Arid	Lower_Arid	-6.139	5.465	**0.047**	-23.77	11.5
		Semi_Arid	-1.016	5.465	**0.04**	-18.65	16.62
		Humid	6.551	5.465	**0.027**	-11.08	24.19
	Lower_Arid	Upper_Arid	6.139	5.465	**0.047**	-11.5	23.77
		Semi_Arid	5.123	6.913	**0.035**	-17.18	27.43
		Humid	12.689	6.913	**0.02**	-9.62	35
	Semi_Arid	Upper_Arid	1.016	5.465	**0.04**	-16.62	18.65
		Lower_Arid	-5.123	6.913	**0.035**	-27.43	17.18
		Humid	7.566	6.913	**0.04**	-14.74	29.87
	Humid	Upper_Arid	-6.551	5.465	**0.027**	-24.19	11.08
		Lower_Arid	-12.689	6.913	**0.02**	-35	9.62
		Semi_Arid	-7.566	6.913	**0.04**	-29.87	14.74
**Armatimonadetes**	Upper_Arid	Lower_Arid	-0.027	0.013	0.441	-0.07	0.01
		Semi_Arid	-0.009	0.013	0.997	-0.05	0.03
		Humid	-0.011	0.013	0.987	-0.05	0.03
	Lower_Arid	Upper_Arid	0.027	0.013	0.441	-0.01	0.07
		Semi_Arid	0.018	0.016	0.952	-0.03	0.07
		Humid	0.016	0.016	0.977	-0.04	0.07
	Semi_Arid	Upper_Arid	0.009	0.013	0.997	-0.03	0.05
		Lower_Arid	-0.018	0.016	0.952	-0.07	0.03
		Humid	-0.002	0.016	1	-0.05	0.05
	Humid	Upper_Arid	0.011	0.013	0.987	-0.03	0.05
		Lower_Arid	-0.016	0.016	0.977	-0.07	0.04
		Semi_Arid	0.002	0.016	1	-0.05	0.05
**Bacteroidetes**	Upper_Arid	Lower_Arid	-1.828	0.901	0.478	-4.73	1.08
		Semi_Arid	-3.99	0.901	**0.002**	-6.9	-1.08
		Humid	-0.908	0.901	0.97	-3.81	2
	Lower_Arid	Upper_Arid	1.828	0.901	0.478	-1.08	4.73
		Semi_Arid	-2.162	1.139	0.562	-5.84	1.51

		Humid	0.92	1.139	0.992	-2.76	4.6
	Semi_Arid	Upper_Arid	3.99	0.901	**0.002**	1.08	6.9
		Lower_Arid	2.162	1.139	0.562	-1.51	5.84
		Humid	3.082	1.139	0.156	-0.59	6.76
	Humid	Upper_Arid	0.908	0.901	0.97	-2	3.81
		Lower_Arid	-0.92	1.139	0.992	-4.6	2.76
		Semi_Arid	-3.082	1.139	0.156	-6.76	0.59
**Chloroflexi**	Upper_Arid	Lower_Arid	-1.421	1.668	0.988	-6.8	3.96
		Semi_Arid	0.212	1.668	1	-5.17	5.59
		Humid	-2.296	1.668	0.861	-7.68	3.08
	Lower_Arid	Upper_Arid	1.421	1.668	0.988	-3.96	6.8
		Semi_Arid	1.632	2.11	0.993	-5.17	8.44
		Humid	-0.875	2.11	1	-7.68	5.93
	Semi_Arid	Upper_Arid	-0.212	1.668	1	-5.59	5.17
		Lower_Arid	-1.632	2.11	0.993	-8.44	5.17
		Humid	-2.508	2.11	0.93	-9.31	4.3
	Humid	Upper_Arid	2.296	1.668	0.861	-3.08	7.68
		Lower_Arid	0.875	2.11	1	-5.93	7.68
		Semi_Arid	2.508	2.11	0.93	-4.3	9.31
**Cyanobacteria**	Upper_Arid	Lower_Arid	6.312	5.512	**0.042**	-11.47	24.1
		Semi_Arid	2.92	5.512	**0.039**	-14.87	20.71
		Humid	-29.446	5.512	**0**	-47.23	-11.66
	Lower_Arid	Upper_Arid	-6.312	5.512	**0.042**	-24.1	11.47
		Semi_Arid	-3.392	6.973	**0.04**	-25.89	19.11
		Humid	-35.759	6.973	**0**	-58.26	-13.26
	Semi_Arid	Upper_Arid	-2.92	5.512	**0.039**	-20.71	14.87
		Lower_Arid	3.392	6.973	**0.04**	-19.11	25.89
		Humid	-32.366	6.973	**0.001**	-54.86	-9.87
	Humid	Upper_Arid	29.446	5.512	**0**	11.66	47.23
		Lower_Arid	35.759	6.973	**0**	13.26	58.26

		Semi_Arid	32.366	6.973	**0.001**	9.87	54.86
**Firmicutes**	Upper_Arid	Lower_Arid	0.013	3.831	1	-12.35	12.37
		Semi_Arid	-0.438	3.831	1	-12.8	11.92
		Humid	-0.007	3.831	1	-12.37	12.35
	Lower_Arid	Upper_Arid	-0.013	3.831	1	-12.37	12.35
		Semi_Arid	-0.451	4.846	1	-16.09	15.18
		Humid	-0.02	4.846	1	-15.66	15.61
	Semi_Arid	Upper_Arid	0.438	3.831	1	-11.92	12.8
		Lower_Arid	0.451	4.846	1	-15.18	16.09
		Humid	0.431	4.846	1	-15.2	16.07
	Humid	Upper_Arid	0.007	3.831	1	-12.35	12.37
		Lower_Arid	0.02	4.846	1	-15.61	15.66
		Semi_Arid	-0.431	4.846	1	-16.07	15.2
**Gemmatimonadetes**	Upper_Arid	Lower_Arid	-0.007	0.045	1	-0.15	0.14
		Semi_Arid	-0.009	0.045	1	-0.16	0.14
		Humid	-0.045	0.045	0.972	-0.19	0.1
	Lower_Arid	Upper_Arid	0.007	0.045	1	-0.14	0.15
		Semi_Arid	-0.002	0.057	1	-0.19	0.18
		Humid	-0.038	0.057	0.998	-0.22	0.15
	Semi_Arid	Upper_Arid	0.009	0.045	1	-0.14	0.16
		Lower_Arid	0.002	0.057	1	-0.18	0.19
		Humid	-0.036	0.057	0.998	-0.22	0.15
	Humid	Upper_Arid	0.045	0.045	0.972	-0.1	0.19
		Lower_Arid	0.038	0.057	0.998	-0.15	0.22
		Semi_Arid	0.036	0.057	0.998	-0.15	0.22
**Nitrospirae**	Upper_Arid	Lower_Arid	0	0.03	1	-0.1	0.1
		Semi_Arid	0	0.03	1	-0.1	0.1
		Humid	0	0.03	1	-0.1	0.1
	Lower_Arid	Upper_Arid	0	0.03	1	-0.1	0.1
		Semi_Arid	0	0.038	1	-0.12	0.12

		Humid	0	0.038	1	-0.12	0.12
	Semi_Arid	Upper_Arid	0	0.03	1	-0.1	0.1
		Lower_Arid	0	0.038	1	-0.12	0.12
		Humid	0	0.038	1	-0.12	0.12
	Humid	Upper_Arid	0	0.03	1	-0.1	0.1
		Lower_Arid	0	0.038	1	-0.12	0.12
		Semi_Arid	0	0.038	1	-0.12	0.12
**Alphaproteobacteria**	Upper_Arid	Lower_Arid	-9.907	3.519	**0.012**	-21.26	1.45
		Semi_Arid	-1.966	3.519	**0.039**	-13.32	9.39
		Humid	3.014	3.519	**0.038**	-8.34	14.37
	Lower_Arid	Upper_Arid	9.907	3.519	**0.012**	-1.45	21.26
		Semi_Arid	7.941	4.451	**0.034**	-6.42	22.3
		Humid	12.922	4.451	**0.01**	-1.44	27.28
	Semi_Arid	Upper_Arid	1.966	3.519	**0.039**	-9.39	13.32
		Lower_Arid	-7.941	4.451	**0.034**	-22.3	6.42
		Humid	4.98	4.451	**0.048**	-9.38	19.34
	Humid	Upper_Arid	-3.014	3.519	**0.038**	-14.37	8.34
		Lower_Arid	-12.922	4.451	**0.01**	-27.28	1.44
		Semi_Arid	-4.98	4.451	**0.048**	-19.34	9.38
**Betaproteobacteria**	Upper_Arid	Lower_Arid	6.964	5.381	**0.035**	-10.4	24.33
		Semi_Arid	-1.764	5.381	**0.04**	-19.13	15.6
		Humid	12.924	5.381	**0.027**	-4.44	30.29
	Lower_Arid	Upper_Arid	-6.964	5.381	**0.035**	-24.33	10.4
		Semi_Arid	-8.728	6.806	**0.043**	-30.69	13.23
		Humid	5.961	6.806	**0.026**	-16	27.92
	Semi_Arid	Upper_Arid	1.764	5.381	**0.04**	-15.6	19.13
		Lower_Arid	8.728	6.806	**0.026**	-13.23	30.69
		Humid	14.688	6.806	**0.04**	-7.27	36.65
	Humid	Upper_Arid	-12.924	5.381	**0.027**	-30.29	4.44
		Lower_Arid	-5.961	6.806	**0.036**	-27.92	16

		Semi_Arid	-14.688	6.806	**0.04**	-36.65	7.27
**Deltaproteobacteria**	Upper_Arid	Lower_Arid	-0.413	0.168	0.249	-0.96	0.13
		Semi_Arid	-0.536	0.168	0.055	-1.08	0.01
		Humid	-3.024	0.168	**0**	-3.57	-2.48
	Lower_Arid	Upper_Arid	0.413	0.168	0.249	-0.13	0.96
		Semi_Arid	-0.123	0.213	0.999	-0.81	0.56
		Humid	-2.611	0.213	**0**	-3.3	-1.92
	Semi_Arid	Upper_Arid	0.536	0.168	0.055	-0.01	1.08
		Lower_Arid	0.123	0.213	0.999	-0.56	0.81
		Humid	-2.488	0.213	**0**	-3.17	-1.8
	Humid	Upper_Arid	3.024	0.168	**0**	2.48	3.57
		Lower_Arid	2.611	0.213	**0**	1.92	3.3
		Semi_Arid	2.488	0.213	**0**	1.8	3.17
**Gammaproteobacteria**	Upper_Arid	Lower_Arid	7.249	6.684	0.956	-14.32	28.82
		Semi_Arid	7.338	6.684	0.953	-14.23	28.91
		Humid	12.064	6.684	0.621	-9.5	33.63
	Lower_Arid	Upper_Arid	-7.249	6.684	0.956	-28.82	14.32
		Semi_Arid	0.089	8.455	1	-27.19	27.37
		Humid	4.815	8.455	0.999	-22.47	32.1
	Semi_Arid	Upper_Arid	-7.338	6.684	0.953	-28.91	14.23
		Lower_Arid	-0.089	8.455	1	-27.37	27.19
		Humid	4.726	8.455	0.999	-22.56	32.01
	Humid	Upper_Arid	-12.064	6.684	0.621	-33.63	9.5
		Lower_Arid	-4.815	8.455	0.999	-32.1	22.47
		Semi_Arid	-4.726	8.455	0.999	-32.01	22.56
**Tenericutes**	Upper_Arid	Lower_Arid	0.008	0.01	0.994	-0.03	0.04
		Semi_Arid	-0.048	0.01	**0.001**	-0.08	-0.01
		Humid	0.008	0.01	0.994	-0.03	0.04
	Lower_Arid	Upper_Arid	-0.008	0.01	0.994	-0.04	0.03
		Semi_Arid	-0.056	0.013	**0.003**	-0.1	-0.01

		Humid	0	0.013	1	-0.04	0.04
	Semi_Arid	Upper_Arid	0.048	0.01	**0.001**	0.01	0.08
		Lower_Arid	0.056	0.013	**0.003**	0.01	0.1
		Humid	0.056	0.013	**0.003**	0.01	0.1
	Humid	Upper_Arid	-0.008	0.01	0.994	-0.04	0.03
		Lower_Arid	0	0.013	1	-0.04	0.04
		Semi_Arid	-0.056	0.013	**0.003**	-0.1	-0.01
**TM7**	Upper_Arid	Lower_Arid	-0.753	0.517	0.823	-2.42	0.91
		Semi_Arid	-0.277	0.517	0.999	-1.94	1.39
		Humid	1.127	0.517	0.387	-0.54	2.79
	Lower_Arid	Upper_Arid	0.753	0.517	0.823	-0.91	2.42
		Semi_Arid	0.476	0.653	0.995	-1.63	2.58
		Humid	1.88	0.653	0.109	-0.23	3.99
	Semi_Arid	Upper_Arid	0.277	0.517	0.999	-1.39	1.94
		Lower_Arid	-0.476	0.653	0.995	-2.58	1.63
		Humid	1.405	0.653	0.405	-0.7	3.51
	Humid	Upper_Arid	-1.127	0.517	0.387	-2.79	0.54
		Lower_Arid	-1.88	0.653	0.109	-3.99	0.23
		Semi_Arid	-1.405	0.653	0.405	-3.51	0.7
**Verrucomicrobia**	Upper_Arid	Lower_Arid	-1.317	0.215	**0**	-2.01	-0.62
		Semi_Arid	-0.602	0.215	0.128	-1.3	0.09
		Humid	-0.895	0.215	**0.004**	-1.59	-0.2
	Lower_Arid	Upper_Arid	1.317	0.215	**0**	0.62	2.01
		Semi_Arid	0.715	0.272	0.181	-0.16	1.59
		Humid	0.422	0.272	0.774	-0.46	1.3
	Semi_Arid	Upper_Arid	0.602	0.215	0.128	-0.09	1.3
		Lower_Arid	-0.715	0.272	0.181	-1.59	0.16
		Humid	-0.293	0.272	0.958	-1.17	0.58
	Humid	Upper_Arid	0.895	0.215	**0.004**	0.2	1.59
		Lower_Arid	-0.422	0.272	0.774	-1.3	0.46

		Semi_Arid	0.293	0.272	0.958	-0.58	1.17
**Unassigned**	Upper_Arid	Lower_Arid	-0.05	0.185	1	-0.65	0.55
		Semi_Arid	0.079	0.185	1	-0.52	0.68
		Humid	0.068	0.185	1	-0.53	0.67
	Lower_Arid	Upper_Arid	0.05	0.185	1	-0.55	0.65
		Semi_Arid	0.13	0.234	0.999	-0.63	0.89
		Humid	0.118	0.234	1	-0.64	0.87
	Semi_Arid	Upper_Arid	-0.079	0.185	1	-0.68	0.52
		Lower_Arid	-0.13	0.234	0.999	-0.89	0.63
		Humid	-0.011	0.234	1	-0.77	0.75
	Humid	Upper_Arid	-0.068	0.185	1	-0.67	0.53
		Lower_Arid	-0.118	0.234	1	-0.87	0.64
		Semi_Arid	0.011	0.234	1	-0.75	0.77

Table S4: Pearson's correlation coefficient values for the increased and decreased of each OTU abundance across the four different bioclimatic zones (r < or >0.9) (p <0.05).

**Increased OTUs following increased aridity**							
**#OTU ID**	**Humid_S**	**Semi_Arid_S**	**Upper_Arid_S**	**Lower_Arid_S**	**R value**	**p_value_Corrected**	**Taxonomy**
**966091**	0.6833713	0.98485864	1.81618205	2.58609138	**0.96727838**	**0.03948368**	Actinobacteria
**453616**	0.13399437	0.44218143	0.58790031	1.21488231	**0.92562359**	**0.03388383**	Actinobacteria
**704830**	0.04689803	0.06699719	0.20545804	0.39305016	**0.9052002**	**0.01176917**	Actinobacteria
**237357**	0	0.03349859	0.18312564	0.25012283	**0.9472398**	**0.04999955**	Actinobacteria
**328**	0.02009916	0.05694761	0.09658761	0.17865916	**0.95746565**	**0.01532002**	Actinobacteria
**11439**	0	0.0167493	0.12952789	0.16525973	**0.92102203**	**0.04085578**	Actinobacteria
**166076**	0	0.02679887	0.09323775	0.12506141	**0.97189391**	**0.04416231**	Actinobacteria
**4331731**	0	0.06029747	0.08318817	0.10272902	**0.92051097**	**0.03310778**	Actinobacteria
**130232**	0	0.02009916	0.03629014	0.0960293	**0.90156471**	**0.03042789**	Actinobacteria
**1079481**	0	0.00334986	0.04913127	0.09156282	**0.91343499**	**0.03204699**	Actinobacteria
**4299608**	0	0.03014873	0.03573183	0.0848631	**0.91311076**	**0.02601724**	Actinobacteria
**331**	0	0.02009916	0.05918085	0.06029747	**0.90558861**	**0.02199741**	Actinobacteria
**760229**	0.00669972	0.02009916	0.03629014	0.05359775	**0.99679354**	**0.01568851**	Actinobacteria
**894047**	0	0.01339944	0.02121578	0.05136451	**0.92316136**	**0.01619099**	Actinobacteria
**1125006**	0	0.01339944	0.02568225	0.04689803	**0.98292747**	**0.01529769**	Actinobacteria
**180752**	0	0.00669972	0.02009916	0.04019831	**0.95238095**	**0.01339944**	Actinobacteria
**837092**	0	0.01004958	0.02289071	0.03796507	**0.99218254**	**0.01267363**	Actinobacteria
**73878**	0	0.01339944	0.01842423	0.03796507	**0.95203022**	**0.011892**	Actinobacteria
**38235**	0	0.00334986	0.01842423	0.03349859	**0.94183976**	**0.01155701**	Actinobacteria

**732609**	0	0.01339944	0.02512394	0.02679887	**0.91805766**	**0.00921211**	Actinobacteria
**4479507**	0.00669972	0.01339944	0.01954085	0.02456563	**0.99599826**	**0.00597392**	Actinobacteria
**179312**	0	0.00334986	0.01395775	0.0223324	**0.96532601**	**0.00776051**	Actinobacteria
**12300**	0	0.00334986	0.00949127	0.01339944	**0.98767025**	**0.00463397**	Actinobacteria
**154591**	0	0.00669972	0.00781634	0.0111662	**0.91090047**	**0.00346152**	Actinobacteria
**516593**	0	0.00334986	0.00669972	0.00893296	**0.99183673**	**0.00301487**	Actinobacteria
**297**	0	0.00334986	0.00502479	0.00893296	**0.97966102**	**0.00284738**	Actinobacteria
**1110760**	0	0.00334986	0.00502479	0.00669972	**0.96571429**	**0.00217741**	Actinobacteria
**71214**	0	0.00334986	0.00390817	0.00669972	**0.94089347**	**0.00206575**	Bacteroidetes
**152**	0.02009916	0.02344902	0.05192282	0.08932958	**0.90197353**	**0.02361651**	Chloroflexi
**4777**	0	0.01339944	0.02959042	0.06253071	**0.9517771**	**0.02037831**	Chloroflexi
**833957**	0	0.00669972	0.01395775	0.02456563	**0.98732097**	**0.00809549**	Chloroflexi
**1093610**	0.02009916	0.03684845	0.08988789	0.14069409	**0.95939279**	**0.04148242**	Firmicutes
**4308955**	0	0.00334986	0.01284113	0.01563268	**0.95025617**	**0.00563893**	Firmicutes
**237492**	0	0.05024789	0.16190987	0.39081692	**0.91018582**	**0.01284113**	Alphaproteobacteria
**4402892**	0.02009916	0.09714592	0.11110367	0.19875832	**0.9400904**	**0.04993524**	Alphaproteobacteria
**2545365**	0	0.01004958	0.02791549	0.06029747	**0.9396204**	**0.01987583**	Alphaproteobacteria
**431**	0	0.0167493	0.0223324	0.05583099	**0.91090047**	**0.01730761**	Alphaproteobacteria
**985216**	0	0.0167493	0.0223324	0.05136451	**0.92886668**	**0.01596766**	Alphaproteobacteria
**246217**	0	0.00669972	0.04019831	0.04913127	**0.92473573**	**0.01808924**	Alphaproteobacteria
**1108982**	0	0.01004958	0.0284738	0.04243155	**0.98876551**	**0.01457189**	Alphaproteobacteria
**64293**	0	0.00334986	0.0167493	0.03796507	**0.91008403**	**0.01272947**	Alphaproteobacteria
**12655**	0.00669972	0.01004958	0.02065747	0.02903211	**0.96532601**	**0.00776051**	Alphaproteobacteria
**926122**	0	0.00334986	0.01786592	0.02679887	**0.95379538**	**0.00949127**	Alphaproteobacteria
**107724**	0	0.00669972	0.01228282	0.0223324	**0.98370198**	**0.00725803**	Alphaproteobacteria
**64271**	0	0.01004958	0.01730761	0.0223324	**0.97756286**	**0.00742552**	Alphaproteobacteria
**334185**	0	0.00669972	0.01451606	0.01563268	**0.93242718**	**0.00547144**	Betaproteobacteria
**566578**	0	0.00334986	0.00390817	0.00893296	**0.91816444**	**0.00273572**	Betaproteobacteria
**3080**	0	0.00669972	0.01898254	0.02679887	**0.98767025**	**0.00926794**	Unassigned

Decreased OTUs following increased aridity

**205635**	0.15409353	0.13399437	0.06253071	0.04689803	**-0.93021021**	**0.03930502**	Acidobacteria
**359**	0.02009916	0.01004958	0.00893296	0.00223324	**-0.91816444**	**0.00547144**	Acidobacteria
**357423**	0.11389522	0.06029747	0.04968958	0.0111662	**-0.94281914**	**0.03187949**	Actinobacteria
**110321**	0.08709634	0.04689803	0.01730761	0.0111662	**-0.91740303**	**0.02573809**	Firmicutes
**154482**	0.05359775	0.02679887	0.01228282	0.00446648	**-0.93465214**	**0.01619099**	Firmicutes
**112836**	0.04689803	0.03349859	0.02679887	0.01786592	**-0.98**	**0.00937961**	Chloroflexi
**316001**	0.01339944	0.00669972	0.00279155	0	**-0.96089299**	**0.00441065**	TM7
**1105689**	0.00669972	0.00334986	0.00223324	0	**-0.96266667**	**0.00212158**	Alphaproteobacteria

Increased OTUs following increased aridity

**#OTU ID**	**Humid_R**	**Semi_Arid_R**	**Upper_Arid_R**	**Lower_Arid_R**	**R value**	**p_value_Corrected**	**Taxonomy**
**509487**	0.02009916	0.08932958	1.98702488	3.00594042	**0.9087046**	**0.04855219**	Actinobacteria
**731014**	0.16749297	0.36178481	0.60129975	1.2595471	**0.91044465**	**0.03515677**	Actinobacteria
**4465539**	0.00669972	0.31488677	0.42487382	1.01612399	**0.91949432**	**0.0313826**	Actinobacteria
**154591**	0	0.02456563	0.07258028	0.17865916	**0.90832683**	**0.05839921**	Actinobacteria
**1110021**	0	0.04913127	0.08151324	0.14069409	**0.98850664**	**0.04544642**	Actinobacteria
**4371107**	0.00669972	0.07816338	0.09770423	0.13846085	**0.94474745**	**0.04148242**	Actinobacteria
**570692**	0	0.03573183	0.07258028	0.10942874	**0.99994389**	**0.03651347**	Actinobacteria
**158859**	0	0.00446648	0.05136451	0.09379606	**0.9197276**	**0.03282862**	Actinobacteria
**249571**	0.01339944	0.04466479	0.06978874	0.0848631	**0.97740779**	**0.02395149**	Actinobacteria
**570552**	0.00669972	0.00893296	0.0452231	0.07146367	**0.91676655**	**0.0230582**	Actinobacteria
**261980**	0.01339944	0.0223324	0.03964	0.06476395	**0.95727998**	**0.01714011**	Actinobacteria

**735771**	0.00669972	0.00893296	0.03461521	0.06253071	**0.90872932**	**0.01931752**	Actinobacteria
**35876**	0	0.02009916	0.03461521	0.05583099	**0.99528001**	**0.0182009**	Actinobacteria
**823633**	0	0.02456563	0.02512394	0.05583099	**0.9004721**	**0.01680513**	Actinobacteria
**244494**	0	0.00223324	0.02568225	0.04019831	**0.92578581**	**0.0144044**	Actinobacteria
**760229**	0	0.00446648	0.01284113	0.02903211	**0.92843308**	**0.0095471**	Actinobacteria
**810563**	0	0.00446648	0.0111662	0.02456563	**0.93913043**	**0.00803966**	Actinobacteria
**698236**	0	0.00446648	0.01004958	0.02009916	**0.96293223**	**0.00658806**	Actinobacteria
**564093**	0	0.00669972	0.01060789	0.01563268	**0.98877612**	**0.00508062**	Actinobacteria
**179312**	0.00669972	0.00893296	0.00893296	0.0111662	**0.9**	**0.00133994**	Actinobacteria
**814193**	0	0.00223324	0.00446648	0.00669972	**1**	**0.00223324**	Actinobacteria
**4299608**	0	0.00223324	0.00334986	0.00669972	**0.96266667**	**0.00212158**	Actinobacteria
**1111326**	0	0.00223324	0.00279155	0.00669972	**0.91571906**	**0.00206575**	Actinobacteria
**810955**	0.02009916	0.06923043	0.09993747	0.22555719	**0.90864452**	**0.04708116**	Bacteroidetes
**4457197**	0	0.05806423	0.07034705	0.1071955	**0.93933281**	**0.03338693**	Bacteroidetes
**255448**	0	0.01786592	0.02400733	0.03349859	**0.95163689**	**0.01066372**	Bacteroidetes
**1119093**	0	0.03796507	0.06253071	0.13846085	**0.94437279**	**0.04399482**	Chloroflexi
**1139057**	0.04019831	0.05136451	0.09993747	0.11166198	**0.92613188**	**0.0262964**	Chloroflexi
**557211**	0	0.00223324	0.01004958	0.01563268	**0.96232465**	**0.00547144**	Cyanobacteria
**1076316**	0	0.00223324	0.0167493	0.03349859	**0.91972258**	**0.01150118**	Firmicutes
**373**	0	0.00223324	0.01395775	0.01786592	**0.93154134**	**0.00653223**	Firmicutes
**2429421**	0	0.00223324	0.00614141	0.00669972	**0.93620253**	**0.00240073**	Firmicutes
**965129**	0.95136004	1.62579838	1.72796909	2.73795167	**0.91549904**	**0.04619456**	Alphaproteobacteria

**1025949**	0	0.06253071	0.13287775	0.33051945	**0.91440921**	**0.01061905**	Alphaproteobacteria
**243360**	0	0.0111662	0.04298986	0.08039662	**0.953072**	**0.02730135**	Alphaproteobacteria
**1003206**	0.00669972	0.03126535	0.04019831	0.06476395	**0.9716763**	**0.01831256**	Alphaproteobacteria
**451696**	0	0.02903211	0.03014873	0.05583099	**0.91003792**	**0.01686096**	Alphaproteobacteria
**4330207**	0	0.01339944	0.01451606	0.03126535	**0.91513616**	**0.00949127**	Alphaproteobacteria
**362799**	0	0.01339944	0.01619099	0.02903211	**0.94966111**	**0.00898879**	Alphaproteobacteria
**64271**	0	0.00223324	0.01004958	0.01786592	**0.95275591**	**0.00614141**	Alphaproteobacteria
**817221**	0	0.00446648	0.00781634	0.01786592	**0.9372973**	**0.00569476**	Alphaproteobacteria
**4348101**	0	0.00669972	0.0111662	0.01563268	**0.98878505**	**0.00513645**	Alphaproteobacteria
**813938**	0	0.00446648	0.00837465	0.01563268	**0.97942046**	**0.00508062**	Alphaproteobacteria
**816760**	0	0.00446648	0.00949127	0.0111662	**0.96474164**	**0.00385234**	Alphaproteobacteria
**345110**	0	0.00223324	0.00446648	0.00893296	**0.96571429**	**0.00290321**	Alphaproteobacteria
**359764**	0	0.03126535	0.06811381	0.0736969	**0.93460023**	**0.02579392**	Betaproteobacteria
**102441**	0	0.00669972	0.01339944	0.02009916	**1**	**0.00669972**	Betaproteobacteria
**78714**	0	0.00669972	0.01284113	0.01786592	**0.99599826**	**0.00597392**	Betaproteobacteria
**844856**	0	0.05583099	0.08932958	0.20769128	**0.9328297**	**0.04656572**	TM7
**251050**	0	0.02903211	0.04968958	0.1071955	**0.9505078**	**0.0342244**	TM7
**88755**	0	0.00893296	0.06085578	0.0736969	**0.91626018**	**0.02730135**	TM7
**111968**	0	0.00223324	0.00837465	0.01563268	**0.95452142**	**0.00530394**	TM7
**Decreased OTUs following increased aridity**							
**278437**	0.02679887	0.01786592	0.01451606	0	**-0.94142259**	**-0.00837465**	Acidobacteria
**850971**	3.9595337	2.14837643	0.76655947	0.46228058	**-0.92292532**	**-0.04873576**	Actinobacteria

**529762**	0.22109071	0.1071955	0.01339944	0.00223324	**-0.9085466**	**-0.04750368**	Actinobacteria
13074	0.15409353	0.1183617	0.05192282	0.01786592	-0.98261365	-0.04751217	Actinobacteria
23	0.12059494	0.08262986	0.0055831	0	-0.91685613	-0.04388316	Actinobacteria
535678	0.06029747	0.03573183	0.00725803	0.00669972	-0.90194247	-0.01892671	Actinobacteria
555495	0.04689803	0.0223324	0.0055831	0.00223324	-0.90875093	-0.01507437	Actinobacteria
437	0.04019831	0.02456563	0.02177409	0	-0.92686213	-0.01233865	Bacteroidetes
24999	0.02009916	0.01563268	0.01060789	0	-0.95227826	-0.00653223	Bacteroidetes
160268	0.11389522	0.06476395	0.00055831	0	-0.90153432	-0.04058913	Chloroflexi
432284	0.0736969	0.04913127	0.04019831	0.01339944	-0.96914822	-0.01898254	Cyanobacteria
592849	0.15409353	0.10496226	0.00949127	0	-0.92461008	-0.04557752	Firmicutes
404204	0.00669972	0.00446648	0.00167493	0	-0.99174041	-0.00228907	Firmicutes
153544	0.00669972	0.00446648	0.00055831	0	-0.93620253	-0.00240073	Firmicutes
21	0.00669972	0.00446648	0.00334986	0.00223324	-0.96571429	-0.00145161	Firmicutes
23204	0.02679887	0.01786592	0.00111662	0	-0.91689885	-0.00971459	Alphaproteobacteria
142233	0.03349859	0.02456563	0.0055831	0.00223324	-0.93975127	-0.01127786	Verrucomicrobia

Table S5: OTUs interaction parameters of each bacterial bioclimatic network such as: Average Shortest Path Length. betweenness centrality. closeness centrality. clustering coefficient. degree. eccentricity. neighborhood connectivity. number of directed edges. radiality. Stress. and Topological Coefficient.

name	SUID	Attribute	Average-Shortest-Path-Length	Betweenness-Centrality	Closeness-Centrality	Clustering-Coefficient	Degree	Eccentricity	Neighborhood-Connectivity	Number-Of-Directed-Edges	Radiality	Stress	Topological-Coefficient	Weight
**570692_R**	125	2	1.11851852	0.16714079	0.89403974	0.22924164	123	4	29.6666667	123	0.98024691	24926	0.22474747	3
**1110021_R**	140	2	1.28888889	0.05905085	0.77586207	0.27980198	101	4	32.1683168	101	0.95185185	15132	0.24555967	3
**Fe**	76	19	1.38518519	0.02658409	0.72192513	0.33452503	89	4	34.6741573	89	0.93580247	7162	0.26672429	5
**328_S**	177	2	1.38518519	0.02591982	0.72192513	0.32865169	89	4	34.3707865	89	0.93580247	7850	0.26439067	1
**251050_R**	93	16	1.40740741	0.02304479	0.71052632	0.32038304	86	4	33.5581395	86	0.93209877	7148	0.25813953	3
**966091_S**	166	2	1.40740741	0.02697123	0.71052632	0.33515732	86	4	34.7441861	86	0.93209877	7380	0.26726297	1
**1093610_S**	160	8	1.42222222	0.02358684	0.703125	0.3471027	84	4	35.2380952	84	0.92962963	6072	0.27106227	1
**Humidity**	75	19	1.42222222	0.0428025	0.703125	0.25888922	83	4	30.1686747	83	0.92962963	11638	0.23029523	5
**1119093_R**	114	5	1.45185185	0.01903204	0.68877551	0.33395062	81	4	33.7037037	81	0.92469136	6110	0.26126902	3
**166076_S**	183	2	1.43703704	0.04361116	0.69587629	0.23888889	81	4	28.7160494	81	0.92716049	11430	0.21920648	1
**13074_R**	91	2	1.47407407	0.0385079	0.67839196	0.23263158	76	4	28.881579	76	0.92098765	9726	0.22047007	4
**844856_R**	92	16	1.51851852	0.01464108	0.65853659	0.36350877	76	5	34.8289474	76	0.91358025	4382	0.27210115	3
**K**	77	19	1.51851852	0.01324416	0.65853659	0.38558559	75	5	36.1466667	75	0.91358025	4082	0.28020672	5
**1025949_R**	108	11	1.56296296	0.01115684	0.63981043	0.38752515	71	5	35.6760563	71	0.90617284	3472	0.28091383	3
**4402892_S**	152	11	1.52592593	0.02256583	0.65533981	0.31871227	71	4	32.1549296	71	0.91234568	6390	0.24926302	1
**453616_S**	173	2	1.56296296	0.01191591	0.63981043	0.39315895	71	5	35.8169014	71	0.90617284	3574	0.28202285	1

**154591_R**	138	2	1.57777778	0.0099346	0.63380282	0.40414079	70	5	36.5285714	70	0.9037037	2984	0.2899093	3
**237492_S**	156	11	1.57037037	0.01020596	0.63679245	0.38881988	70	5	35.8714286	70	0.90493827	3126	0.28245219	1
**4465539_R**	129	2	1.58518519	0.01095042	0.63084112	0.40781387	68	5	36.2794118	68	0.90246914	3188	0.28566466	3
**704830_S**	171	2	1.58518519	0.00899566	0.63084112	0.42062415	67	5	37.8208955	67	0.90246914	2838	0.29547575	1
**965129_R**	97	11	1.59259259	0.01094852	0.62790698	0.39938556	63	4	35.4603175	63	0.90123457	3412	0.27703373	3
**357423_S**	142	2	1.62962963	0.01626247	0.61363636	0.28508772	57	4	29.5263158	57	0.89506173	4056	0.22888617	2
**509487_R**	128	2	1.66666667	0.00814318	0.6	0.39415584	56	5	35.6964286	56	0.88888889	2040	0.27887835	3
**4371107_R**	130	2	1.6	0.04133453	0.625	0.17792208	56	3	23.125	56	0.9	7074	0.17387218	3
**4457197_R**	117	4	1.65185185	0.01889057	0.60538117	0.185884	54	4	24.0185185	54	0.89135802	3688	0.18619007	3
**1139057_R**	113	5	1.6962963	0.0114413	0.58951965	0.33632653	50	4	29.54	50	0.88395062	3448	0.23259843	3
**205635_S**	141	1	1.72592593	0.00822368	0.57939914	0.34975845	46	4	31.0652174	46	0.87901235	3158	0.24460801	2
**11439_S**	188	2	1.71851852	0.00890737	0.58189655	0.29178744	46	4	30.4782609	46	0.88024691	2376	0.23811141	1
**592849_R**	85	8	1.75555556	0.00873332	0.56962025	0.30853659	41	4	26.6097561	41	0.87407407	2480	0.20788872	4
**23_R**	90	2	1.8	0.00568314	0.55555556	0.36031746	36	4	27.9166667	36	0.86666667	1662	0.21981627	4
**160268_R**	88	5	1.97777778	0.01015813	0.50561798	0.35698925	31	3	24.3870968	31	0.83703704	2468	0.22791679	4
**102441_R**	96	12	1.83703704	0.00134455	0.54435484	0.54942529	30	4	70.7666667	30	0.86049383	952	0.55286458	3
**814193_R**	119	2	1.83703704	0.00134455	0.54435484	0.54942529	30	4	70.7666667	30	0.86049383	952	0.55286458	3
**760229_S**	169	2	1.83703704	0.00134455	0.54435484	0.54942529	30	4	70.7666667	30	0.86049383	952	0.55286458	1
**837092_S**	168	2	1.85185185	8.56E-04	0.54	0.5978836	28	4	73.0714286	28	0.85802469	700	0.57087054	1
**833957_S**	161	5	1.86666667	5.53E-04	0.53571429	0.64615385	26	4	75	26	0.85555556	462	0.5859375	1
**1125006_S**	190	2	1.87407407	4.37E-04	0.53359684	0.67666667	25	4	76.16	25	0.85432099	374	0.595	1
**Phosphate**	74	19	2.20740741	0.04961746	0.45302013	0.11594203	24	4	12.25	24	0.79876543	37258	0.14583333	5
**813938_R**	100	11	1.88148148	3.61E-04	0.53149606	0.69202899	24	4	77	24	0.85308642	340	0.6015625	3

**35876_R**	132	2	1.88148148	3.61E-04	0.53149606	0.69202899	24	4	77	24	0.85308642	340	0.6015625	3
**1108982_S**	158	11	1.91111111	5.87E-04	0.52325581	0.64492754	24	5	73.0833333	24	0.84814815	462	0.57545932	1
**107724_S**	159	11	1.88148148	3.61E-04	0.53149606	0.69202899	24	4	77	24	0.85308642	340	0.6015625	1
**4299608_R**	131	2	1.88888889	3.09E-04	0.52941176	0.71936759	23	4	76.826087	23	0.85185185	282	0.6002038	3
**111968_R**	94	16	1.91851852	3.30E-04	0.52123552	0.71146245	23	5	75.8695652	23	0.84691358	352	0.59739815	3
**64271_R**	101	11	1.91851852	3.30E-04	0.52123552	0.71146245	23	5	75.8695652	23	0.84691358	352	0.59739815	3
**345110_R**	106	11	1.91851852	2.18E-04	0.52123552	0.75889328	23	5	78	23	0.84691358	206	0.61417323	3
**243360_R**	107	11	1.91851852	3.30E-04	0.52123552	0.71146245	23	5	75.8695652	23	0.84691358	352	0.59739815	3
**557211_R**	112	6	1.91851852	5.04E-04	0.52123552	0.64822134	23	5	73.173913	23	0.84691358	396	0.57617254	3
**261980_R**	133	2	1.91851852	2.41E-04	0.52123552	0.75098814	23	5	77.5217391	23	0.84691358	244	0.61040739	3
**12655_S**	157	11	1.91851852	4.16E-04	0.52123552	0.67588933	23	5	74.6956522	23	0.84691358	384	0.58815474	1
**38235_S**	176	2	1.91851852	3.30E-04	0.52123552	0.71146245	23	5	75.8695652	23	0.84691358	352	0.59739815	1
**297_S**	178	2	1.88888889	3.29E-04	0.52941176	0.68774704	23	4	77.4347826	23	0.85185185	318	0.60495924	1
**180752_S**	180	2	1.91851852	2.41E-04	0.52123552	0.75098814	23	5	77.5217391	23	0.84691358	244	0.61040739	1
**179312_S**	181	2	1.91851852	4.16E-04	0.52123552	0.67588933	23	5	74.6956522	23	0.84691358	384	0.58815474	1
**78714_R**	95	12	1.8962963	9.94E-04	0.52734375	0.51948052	22	4	70.1363636	22	0.85061728	1016	0.54794034	3
**24999_R**	81	4	1.92592593	1.60E-04	0.51923077	0.79220779	22	5	78.9545455	22	0.84567901	174	0.62168933	4
**810563_R**	120	2	1.92592593	1.60E-04	0.51923077	0.79220779	22	5	78.9545455	22	0.84567901	174	0.62168933	3
**698236_R**	124	2	1.92592593	1.60E-04	0.51923077	0.79220779	22	5	78.9545455	22	0.84567901	174	0.62168933	3
**2545365_S**	154	11	1.92592593	1.60E-04	0.51923077	0.79220779	22	5	78.9545455	22	0.84567901	174	0.62168933	1
**4777_S**	162	5	1.92592593	1.60E-04	0.51923077	0.79220779	22	5	78.9545455	22	0.84567901	174	0.62168933	1
**4479507_S**	174	2	1.8962963	9.94E-04	0.52734375	0.51948052	22	4	70.1363636	22	0.85061728	1016	0.54794034	1
**4348101_R**	103	11	1.88148148	0.00577716	0.53149606	0.5	21	3	69.4285714	21	0.85308642	6550	0.53406593	3

**432284_R**	80	6	1.9037037	2.30E-04	0.52529183	0.74761905	21	4	77	21	0.84938272	206	0.6015625	4
**278437_R**	79	1	1.91851852	2.09E-04	0.52123552	0.79047619	21	4	76.5714286	21	0.84691358	168	0.60770975	4
**1076316_R**	111	8	1.93333333	1.63E-04	0.51724138	0.78095238	21	5	78.5238095	21	0.84444444	190	0.61829771	3
**760229_R**	121	2	1.93333333	1.18E-04	0.51724138	0.82857143	21	5	79.0952381	21	0.84444444	124	0.62279715	3
**158859_R**	137	2	1.93333333	2.39E-04	0.51724138	0.71904762	21	5	76.4761905	21	0.84444444	260	0.60217473	3
**3080_S**	146	18	1.9037037	7.36E-04	0.52529183	0.63809524	21	4	71.2857143	21	0.84938272	744	0.55691964	1
**926122_S**	149	11	1.93333333	3.94E-04	0.51724138	0.66190476	21	5	72.6666667	21	0.84444444	292	0.57217848	1
**152_S**	163	5	1.92592593	0.01494232	0.51923077	0.73809524	21	5	74.952381	21	0.84567901	3444	0.58980127	1
**73878_S**	170	2	1.91851852	2.09E-04	0.52123552	0.79047619	21	4	76.5714286	21	0.84691358	168	0.60770975	1
**12300_S**	186	2	1.9037037	7.36E-04	0.52529183	0.63809524	21	4	71.2857143	21	0.84938272	744	0.55691964	1
**1079481_S**	193	2	1.93333333	2.39E-04	0.51724138	0.71904762	21	5	76.4761905	21	0.84444444	260	0.60217473	1
**1003206_R**	109	11	1.9037037	0.01499539	0.52529183	0.66842105	20	4	73.9	20	0.84938272	2662	0.57695312	3
**564093_R**	127	2	1.91111111	3.68E-04	0.52325581	0.63157895	20	4	76.8	20	0.84814815	298	0.6	3
**735771_R**	122	2	1.94074074	1.38E-04	0.51526718	0.78421053	20	5	79.05	20	0.84320988	164	0.62244094	3
**985216_S**	148	11	1.95555556	1.04E-04	0.51136364	0.84210526	20	5	77.6	20	0.84074074	60	0.6208	1
**516593_S**	172	2	1.91111111	7.50E-04	0.52325581	0.53157895	20	4	69.3	20	0.84814815	786	0.54140625	1
**404204_R**	86	8	1.91851852	6.37E-04	0.52123552	0.56140351	19	4	69.7368421	19	0.84691358	670	0.54481908	4
**431_S**	153	11	1.96296296	4.82E-05	0.50943396	0.90643275	19	5	78.8421053	19	0.83950617	32	0.63073684	1
**64293_S**	150	11	1.94814815	7.25E-05	0.51330798	0.86549708	19	5	80.3684211	19	0.84197531	90	0.63282221	1
**437_R**	78	4	1.94074074	1.44E-04	0.51526718	0.81045752	18	4	76.1666667	18	0.84320988	128	0.60449735	4
**817221_R**	98	11	1.97037037	1.71E-05	0.5075188	0.95424837	18	5	80.0555556	18	0.8382716	14	0.64044444	3
**810955_R**	116	4	1.97037037	1.71E-05	0.5075188	0.95424837	18	5	80.0555556	18	0.8382716	14	0.64044444	3
**731014_R**	123	2	1.97037037	1.71E-05	0.5075188	0.95424837	18	5	80.0555556	18	0.8382716	14	0.64044444	3

**570552_R**	126	2	1.95555556	1.62E-04	0.51136364	0.73202614	18	5	74.8888889	18	0.84074074	168	0.58967629	3
**1111326_R**	139	2	1.97037037	7.89E-05	0.5075188	0.85620915	18	5	76.7777778	18	0.8382716	44	0.61422222	3
**566578_S**	147	12	1.94074074	1.44E-04	0.51526718	0.81045752	18	4	76.1666667	18	0.84320988	128	0.60449735	1
**894047_S**	167	2	1.97037037	1.71E-05	0.5075188	0.95424837	18	5	80.0555556	18	0.8382716	14	0.64044444	1
**130232_S**	184	2	1.97037037	1.71E-05	0.5075188	0.95424837	18	5	80.0555556	18	0.8382716	14	0.64044444	1
**4330207_R**	104	11	1.94814815	1.20E-04	0.51330798	0.81617647	17	4	76.5294118	17	0.84197531	110	0.60737628	3
**4299608_S**	175	2	1.97777778	6.18E-05	0.50561798	0.86764706	17	5	77.3529412	17	0.83703704	36	0.61882353	1
**244494_R**	135	2	1.97037037	1.08E-04	0.5075188	0.76666667	16	5	74.4375	16	0.8382716	104	0.58612205	3
**237357_S**	179	2	1.97037037	1.48E-04	0.5075188	0.74166667	16	5	72.125	16	0.8382716	102	0.56791339	1
**816760_R**	99	11	1.92592593	0.00254151	0.51923077	0.55238095	15	3	62.2666667	15	0.84567901	3544	0.47897436	3
**249571_R**	134	2	1.92592593	0.00238931	0.51923077	0.54285714	15	3	63	15	0.84567901	3434	0.48461538	3
**362799_R**	105	11	1.96296296	8.35E-05	0.50943396	0.81904762	15	4	77.0666667	15	0.83950617	78	0.61164021	3
**823633_R**	118	2	2.07407407	9.11E-05	0.48214286	0.79120879	14	4	72.7857143	14	0.82098765	88	0.64987245	3
**112836_S**	2155	5	1.93333333	0.00346327	0.51724138	0.61538462	14	3	75.5714286	14	0.84444444	4206	0.58131868	2
**64271_S**	151	11	1.93333333	0.00202437	0.51724138	0.51648352	14	3	62.8571429	14	0.84444444	3030	0.48351648	1
**21_R**	2149	8	1.93333333	0.0165442	0.51724138	0.43589744	13	3	61.4615385	13	0.84444444	4230	0.47218935	4
**88755_R**	2150	16	1.97777778	1.24E-04	0.50561798	0.80769231	13	4	64.5384615	13	0.83703704	140	0.51221001	3
**359764_R**	2151	12	1.93333333	0.016483	0.51724138	0.53846154	13	3	53.3846154	13	0.84444444	3632	0.41005917	3
**373_R**	110	8	2.00740741	6.18E-05	0.49815498	0.82051282	13	5	69	13	0.83209877	28	0.552	3
**71214_S**	164	4	1.97777778	5.60E-05	0.50561798	0.83333333	13	4	76.6153846	13	0.83703704	56	0.60805861	1
**451696_R**	102	11	1.98518519	4.73E-05	0.50373134	0.84848485	12	4	75.5833333	12	0.83580247	50	0.59986772	3
**179312_R**	136	2	2.08888889	5.60E-05	0.4787234	0.8030303	12	4	72.75	12	0.81851852	56	0.64955357	3
**1110760_S**	191	2	1.94814815	0.00172939	0.51330798	0.51515152	12	3	66.5	12	0.84197531	2728	0.51153846	1

**142233_R**	82	17	1.99259259	9.93E-05	0.50185874	0.81818182	11	4	67.9090909	11	0.8345679	130	0.53896104	4
**1105689_S**	2154	11	1.99259259	3.26E-05	0.50185874	0.83636364	11	4	77.1818182	11	0.8345679	28	0.61255411	2
**2429421_R**	2152	8	2	4.72E-05	0.5	0.86666667	10	4	61.3	10	0.83333333	58	0.48650794	3
**23204_R**	84	11	2.00740741	2.21E-05	0.49815498	0.97222222	9	4	63	9	0.83209877	48	0.5	4
**153544_R**	87	8	2.00740741	2.21E-05	0.49815498	0.97222222	9	4	63	9	0.83209877	48	0.5	4
**535678_R**	89	2	2.20740741	9.88E-04	0.45302013	0.80555556	9	3	52	9	0.79876543	1490	0.53061224	4
**316001_S**	2153	16	1.97037037	0.0013008	0.5075188	0.58333333	9	3	72.2222222	9	0.8382716	2074	0.55555556	2
**246217_S**	155	11	2.03703704	3.16E-06	0.49090909	0.97222222	9	5	77.5555556	9	0.82716049	2	0.62044444	1
**4308955_S**	2158	8	2.03703704	3.16E-06	0.49090909	0.97222222	9	5	77.5555556	9	0.82716049	2	0.62044444	1
**850971_R**	2143	2	1.97777778	7.16E-04	0.50561798	0.5	8	3	55.125	8	0.83703704	1104	0.42403846	4
**555495_R**	2144	2	1.97777778	7.16E-04	0.50561798	0.5	8	3	55.125	8	0.83703704	1104	0.42403846	4
**255448_R**	115	4	1.97777778	0.0010201	0.50561798	0.64285714	8	3	70.25	8	0.83703704	1620	0.54038462	3
**529762_R**	2145	2	1.98518519	6.26E-04	0.50373134	0.57142857	7	3	55.2857143	7	0.83580247	1008	0.42527473	4
**359_S**	143	1	2.02222222	0	0.49450549	1	7	4	75	7	0.82962963	0	0.5952381	2
**4331731_S**	145	2	1.98518519	7.88E-04	0.50373134	0.57142857	7	3	63.7142857	7	0.83580247	1284	0.49010989	1
**334185_S**	2157	12	1.98518519	0.00116501	0.50373134	0.57142857	7	3	66.1428571	7	0.83580247	1696	0.50879121	1
**154591_S**	165	2	1.98518519	8.53E-04	0.50373134	0.76190476	7	3	69.4285714	7	0.83580247	1336	0.53406593	1
**154482_S**	2148	8	1.99259259	6.70E-04	0.50185874	0.73333333	6	3	69.1666667	6	0.8345679	1084	0.53205128	2
**331_S**	2160	2	2.06666667	0	0.48387097	1	5	5	76.6	5	0.82222222	0	0.6128	1
**110321_S**	2147	8	2.00740741	3.33E-04	0.49815498	0.66666667	4	3	64.25	4	0.83209877	552	0.49423077	2
**732609_S**	2159	2	2.00740741	4.95E-04	0.49815498	0.5	4	3	71	4	0.83209877	862	0.54615385	1
**CEC**	73	19	3.17777778	0.00735213	0.31468531	0.66666667	3	5	9.66666667	3	0.63703704	9164	0.38666667	5
**Nitrogen**	72	19	3.17777778	0.00735213	0.31468531	0.66666667	3	5	9.66666667	3	0.63703704	9164	0.38666667	5

**Organic**	2142	19	4.16296296	0	0.24021352	1	2	6	3	2	0.47283951	0	1	5
**79539_R**	83	13	2.92592593	0	0.34177215	0	1	4	13	1	0.67901235	0	0	4
**538093_R**	2146	2	2.8962963	0	0.34526854	0	1	5	20	1	0.68395062	0	0	3
**1110021_S**	144	2	2.92592593	0	0.34177215	0	1	4	13	1	0.67901235	0	0	1
**175954_S**	2156	2	2.91851852	0	0.34263959	0	1	6	21	1	0.68024691	0	0	1

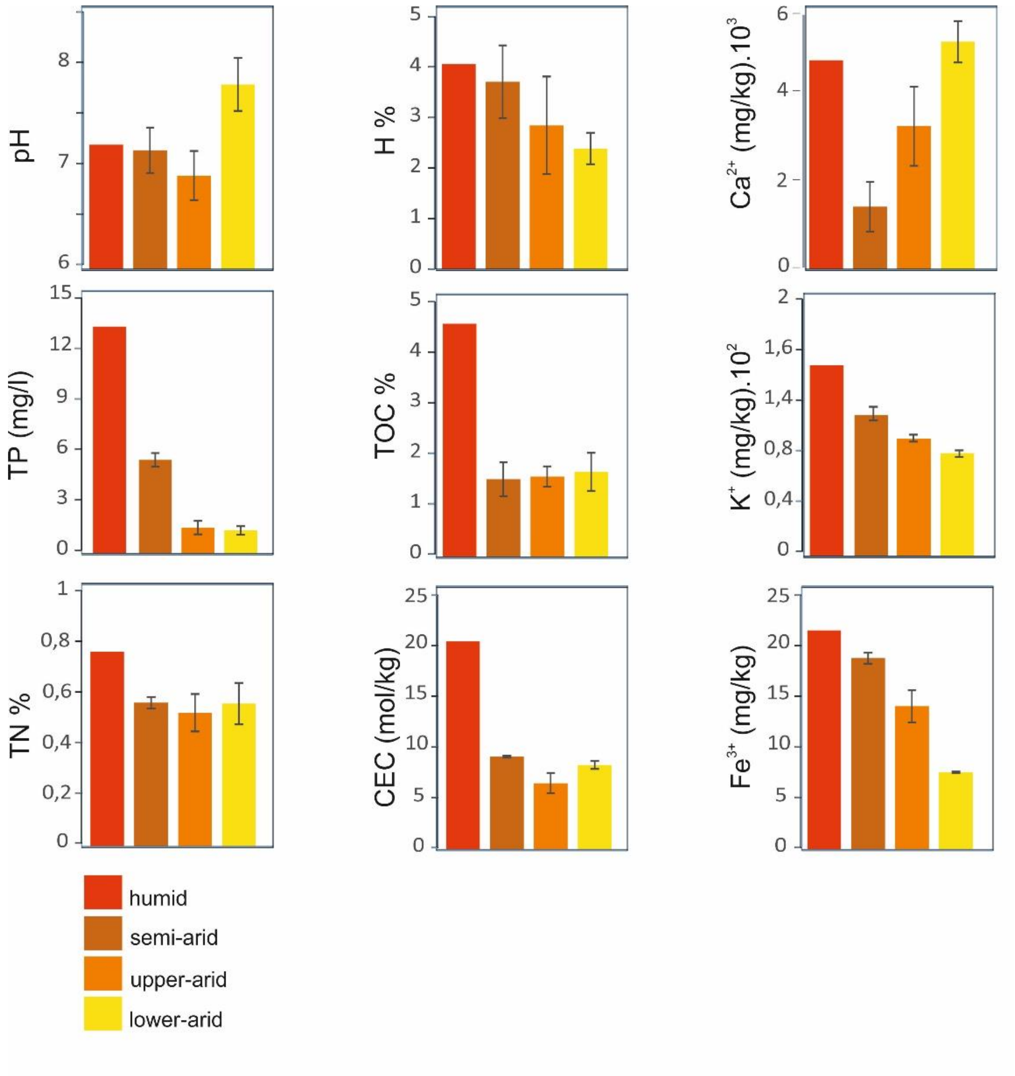

Supplementary Figure 1:

Principal characteristics of soil samples of spineless *Optunia ficus indica* collected across the four bioclimatic stages. Potassium ion (K^+). calcium ion (Ca^{2+}). iron ion (Fe^{3+}). pH. Total Organic Carbon (TOC). Total Nitrogen (TN). Total Phosphorus (TP). Humidity (H). Cation Exchange Capacity (CEC). Different letters symbolize statistically significant difference between means (p <0.05) based on HDS Tukey test.

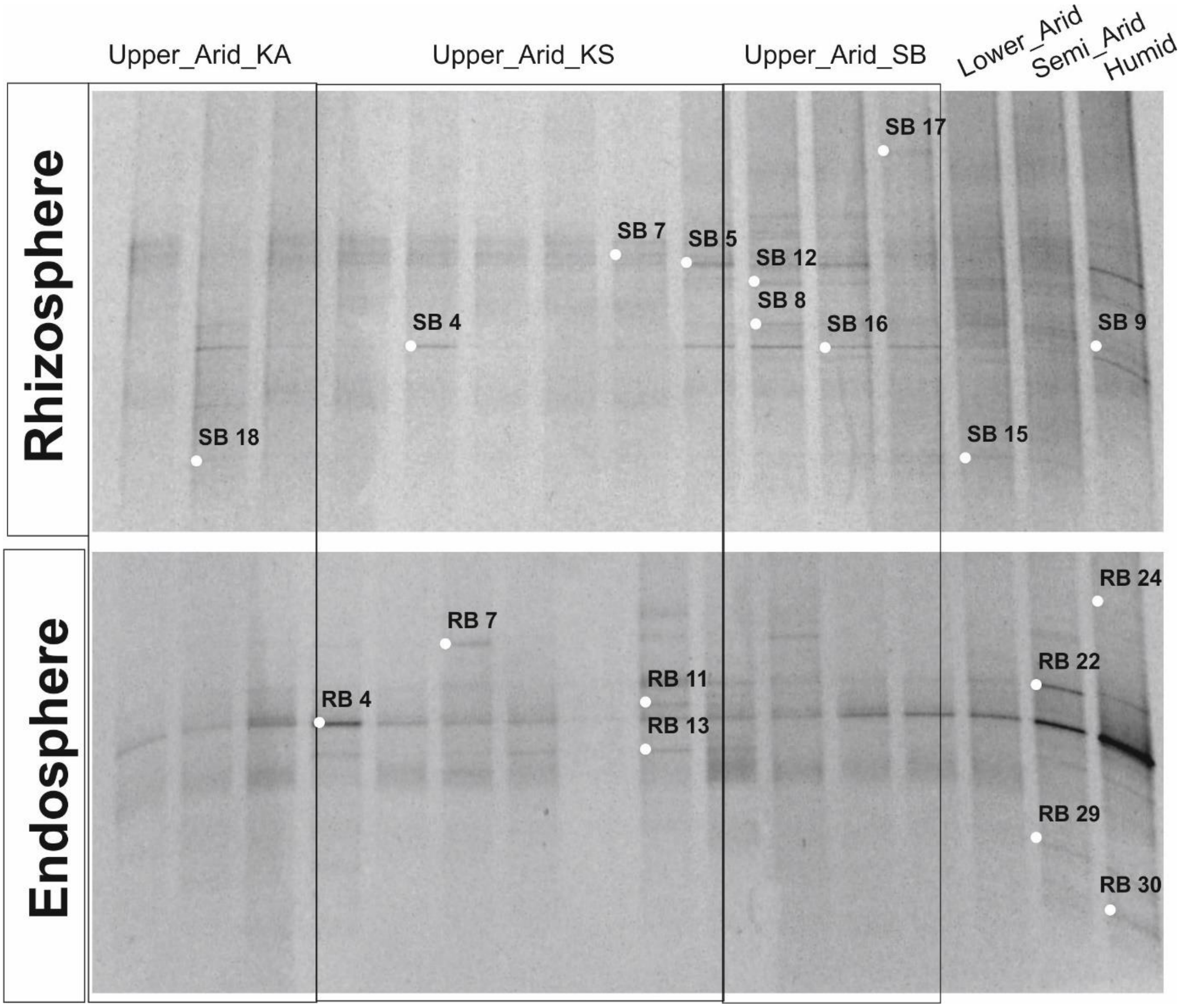

Supplementary Figure 2:

Denaturing gradient gel electrophoresis profiles of PCR-amplified V3-V5 region fragments of bacterial 16S rDNA gene obtained from samples (upper arid KA. upper-arid KS. upper-arid SB. lower-arid. semi-arid and humid) collected from rhizosphere (**a**) and endosphere (**b**). The positions of the dominant bands excised and sequenced from rhizosphere (SB) and endosphere (RB) are indicated on DGGE profiles.

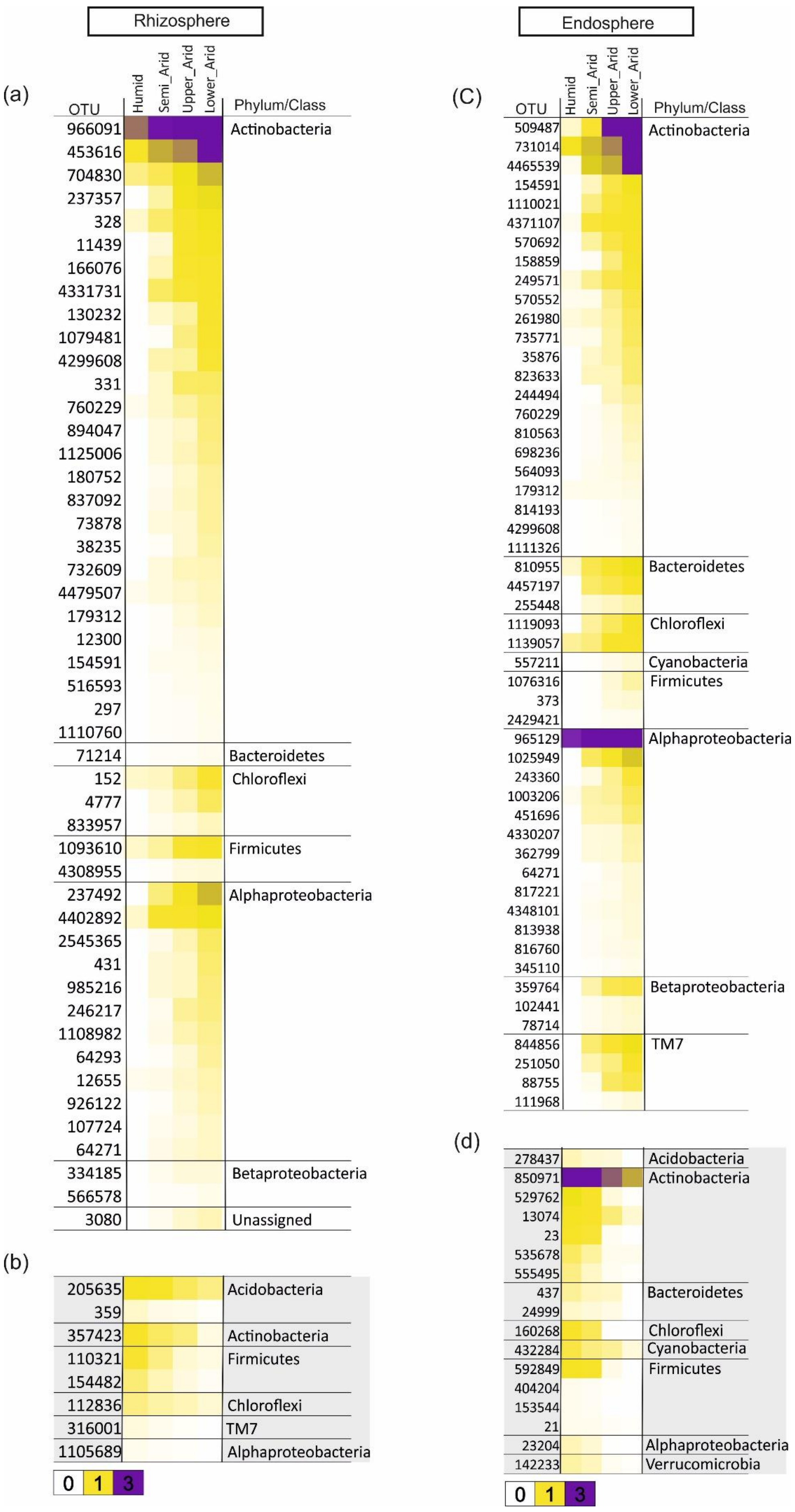
Rhizosphere
Endosphere
(a)
(b)
(C)
(d)
OTU
Humid
Semi_Arid
Upper_Arid
Lower_Arid
Phylum/Class
966091
453616
704830
237357
328
11439
166076
4331731
130232
1079481
4299608
331
760229
894047
1125006
180752
837092
73878
38235
732609
4479507
179312
12300
154591
516593
297
1110760
Actinobacteria
71214
Bacteroidetes
152
4777
833957
Chloroflexi
1093610
4308955
Firmicutes
237492
4402892
2545365
431
985216
246217
1108982
64293
12655
926122
107724
64271
Alphaproteobacteria
334185
566578
Betaproteobacteria
3080
Unassigned
205635
359
Acidobacteria
357423
Actinobacteria
110321
154482
Firmicutes
112836
Chloroflexi
316001
TM7
1105689
Alphaproteobacteria
0
1
3
509487
731014
4465539
154591
1110021
4371107
570692
158859
249571
570552
261980
735771
35876
823633
244494
760229
810563
698236
564093
179312
814193
4299608
1111326
Actinobacteria
810955
4457197
255448
Bacteroidetes
1119093
1139057
Chloroflexi
557211
Cyanobacteria
1076316
373
2429421
Firmicutes
965129
1025949
243360
1003206
451696
4330207
362799
64271
817221
4348101
813938
816760
345110
Alphaproteobacteria
359764
102441
78714
Betaproteobacteria
844856
251050
88755
111968
TM7
278437
Acidobacteria
850971
529762
13074
23
535678
555495
Actinobacteria
437
24999
Bacteroidetes
160268
Chloroflexi
432284
Cyanobacteria
592849
404204
153544
21
Firmicutes
23204
Alphaproteobacteria
142233
Verrucomicrobia
0
1
3

Supplementary Figure 3:

Heat map showing OTUs classified based on their abundances whether increased or decreased following strictly (> or < 0.9) the aridity degree in the endosphere and rhizosphere samples. The relative abundance of dominant and moderate dominant (> or = 0.1% of all sequences) in rhizosphere and endosphere samples across the aridity gradient. Rhizosphere increased OTUs (**a**). rhizosphere decreased (**b**) OTUs. Endosphere increased OTUs (**c**) and endosphere decreased (**d**) OTUs. The OTU ID is noted to the left of the heatmap while the annotation. given as the phylum/ class taxonomic level. is to the right of the heatmap. The color intensity for each panel corresponds to the OTU abundance. white (0%) indicates low relative abundance. through yellow (>1%) to purple (>3%) which indicates a high level of relative abundance.

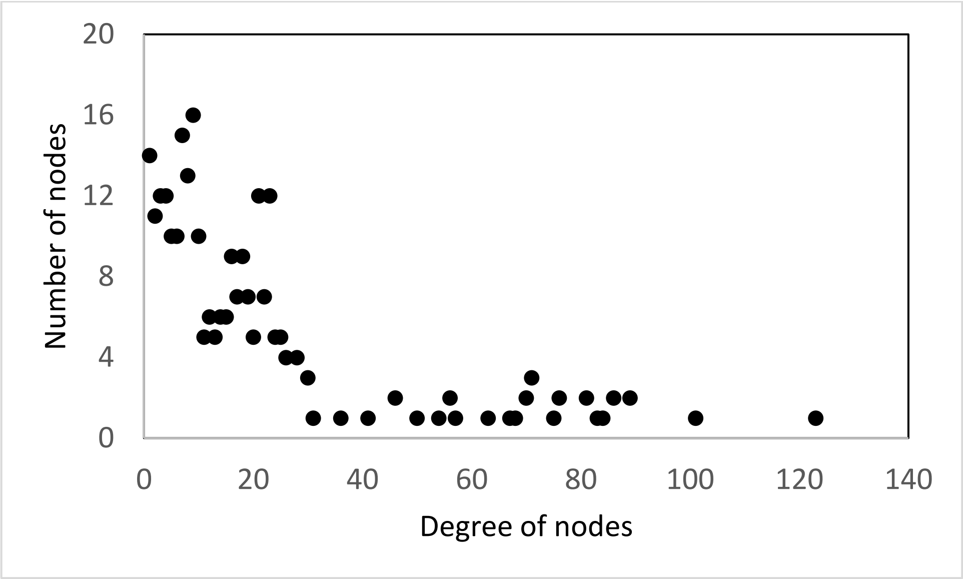

Supplementary Figure 4:

The node degree distribution of scale-free network of the combined two bacterial subnetworks. The X axis is the degree of a node (number of connections between taxa and soil parameters). and the Y axis represents the number of nodes that correspond to the degree.
